# Protein Aggregation Patterns Inform about Breast Cancer Response to Antiestrogens and Reveal the RNA Ligase RTCB as Mediator of Acquired Tamoxifen Resistance

**DOI:** 10.3390/cancers13133195

**Published:** 2021-06-26

**Authors:** Inês Direito, Liliana Monteiro, Tânia Melo, Daniela Figueira, João Lobo, Vera Enes, Gabriela Moura, Rui Henrique, Manuel A. S. Santos, Carmen Jerónimo, Francisco Amado, Margarida Fardilha, Luisa A. Helguero

**Affiliations:** 1 iBiMED—Institute of Biomedicine, University of Aveiro, 3810-193 Aveiro, Portugal; inesgdireito@ua.pt (I.D.); lilianamonteiro@ua.pt (L.M.); dfdfigueira@live.ua.pt (D.F.); vera.enes@ua.pt (V.E.); gmoura@ua.pt (G.M.); msantos@ua.pt (M.A.S.S.); mfardilha@ua.pt (M.F.); 2 LaQV-REQUIMTE—Associated Laboratory for Green Chemistry of the Network of Chemistry and Technology, University of Aveiro, 3810-193 Aveiro, Portugal; taniamelo@ua.pt (T.M.); famado@ua.pt (F.A.); 3 Department of Pathology, Portuguese Oncology Institute of Porto (IPOP), 4200-072 Porto, Portugal; joaomachadolobo@gmail.com (J.L.); rmhenrique@icbas.up.pt (R.H.); carmenjeronimo@ipoporto.min-saude.pt (C.J.); 4 Cancer Biology and Epigenetics Group, IPO Porto Research Center (GEBC CI-IPOP), Portuguese Oncology Institute of Porto (IPO Porto) & Porto Comprehensive Cancer Center (P.CCC), 4200-072 Porto, Portugal; 5 Department of Pathology and Molecular Immunology, Institute of Biomedical Sciences Abel Salazar, University of Porto (ICBAS-UP), Rua Jorge Viterbo Ferreira 228, 4050-513 Porto, Portugal

**Keywords:** breast cancer, estrogen receptors, antiestrogen resistance, protein aggregation, tRNA-splicing ligase RTCB homolog (RTCB)

## Abstract

**Simple Summary:**

Acquired resistance to antiestrogenic therapy remains the major obstacle to curing luminal subtype breast cancer. While current treatment in acquired endocrine-resistant settings includes combined therapy with receptor tyrosine kinase or cyclin-dependent kinase inhibitors, progression to incurable disease remains possible. In recent years, the antioxidant system and the protein quality control network have been associated with the enhanced resistance of breast cancer cells to hormonal therapy. In this work, we raise the hypothesis that antiestrogen treatment induces the accumulation of protein aggregates in sensitive cells, which in turn could hinder the activation of survival pathways. We present evidence concerning a novel way to identify antiestrogen response and disclose a novel protein, RTBC, that controls acquired antiestrogen resistance. This work opens a new avenue for research towards finding breast cancer prognostic markers and therapeutic targets, where the identification of proteins prone to aggregate could help to identify antiestrogen response and understand mechanisms of disease.

**Abstract:**

The protein quality control network, including autophagy, the proteasome and the unfolded protein response (UPR), is triggered by stress and is overactive in acquired antiestrogen therapy resistance. We show for the first time that the aggresome load correlates with apoptosis and is increased in antiestrogen-sensitive cells compared to endocrine-resistant variants. LC-MS/MS analysis of the aggregated proteins obtained after 4OH-tamoxifen and Fulvestrant treatment identified proteins with essential function in protein quality control in antiestrogen-sensitive cells, but not in resistant variants. These include the UPR modulators RTCB and PDIA6, as well as many proteasome proteins such as PSMC2 and PSMD11. RTCB is a tRNA and XBP1 ligase and its aggregation induced by antiestrogens correlated with impaired XBP1s expression in sensitive cells. Knock down of RTCB was sufficient to restore sensitivity to tamoxifen in endocrine-resistant cells and increased the formation of aggresomes, leading to apoptotic cell death. Analysis of primary human breast cancer samples and their metastases appearing after endocrine treatment showed that RTCB is only localized to aggresomes in the primary tumors, while total aggresomes, including aggregated RTCB, were significantly reduced in the metastases. Therefore, different protein aggregation patterns may indicate loss of function of essential proteins resulting in enhanced protein aggregation that can be used to identify antiestrogen-resistant breast cancer cells and improve the response to antiestrogenic therapy.

## 1. Introduction

Development of resistance to endocrine therapy remains the main obstacle for curing estrogen receptor alpha (ERα)-positive breast cancer, with 30–50% of the patients that initially respond progressing to incurable disease [1,2]. Pre-clinical studies have shown that the unfolded protein response (UPR) and autophagy (AUT) are enhanced, allowing breast cancer resistance to antiestrogen therapy [3,4,5,6,7,8,9]. AUT and UPR compensate for therapy-induced stress (i.e., metabolic, oxidative) and mutational load to maintain protein folding and prevent accumulation of toxic protein aggregates [3,10,11,12]. Disruption of proteostasis to improve anti-cancer therapy has been a topic of increasing research in recent years [13,14].

The UPR is initiated in response to the accumulation of misfolded proteins in the lumen of the endoplasmic reticulum (EnR). Upon BIP releasing itself from the EnR-resident proteins (IRE1α, PERK and ATF6) to bind the misfolded proteins, the EnR-resident proteins initiate the three UPR cascades to restore proteostasis by inhibiting translation and increasing chaperoning and EnR capacity [15]. In breast cancer cells, activation of IRE1α branch by estrogens leads to an anticipatory response prior to cell division, and is also enhanced in antiestrogen resistance [16]. Initiation of the IRE1α branch consists of an unconventional cytoplasmic splicing [17], where the IRE1α endoribonuclease domain removes an intron from the XBP1 mRNA followed by exon ligation by RNA-splicing ligase RtcB homolog (RTCB). The resulting XBP1 mRNA is translated into a functional transcription factor [18] that stimulates the expression of EnR chaperones such as BiP, lipid synthesis and EnR-associated protein degradation (ERAD) [15]. XBP1s is also an estrogen-responsive gene [19]; it can function as an ERα transcriptional co-activator, and upregulation of XBP1s is strongly associated with antiestrogen resistance [4,20]. Inhibition of IRE1α activity or autophagy can reduce endocrine resistance in breast cancer [6,21,22], supporting the notion that unresolved protein aggregation could enhance drug toxicity.

Toxic protein aggregates have been extensively linked to neurodegenerative diseases [23,24], but their significance has not been followed-up in cancer, with few exceptions, such as mutant p53, which undergoes prion-like aggregation to induce loss of wild-type tumor suppression function [25]. This supports the idea that protein aggregation induced by anti-cancer treatment can engage essential survival proteins, leading to their loss of function. Recently, HDAC6 inhibitors were shown to enhance sensitivity to radiation of breast cancer cell lines by increasing protein aggregation [26]. In this work, we aimed to characterize the aggregated proteome and identify essential proteins needed for endocrine-resistance. We show for the first time, that protein aggregation patterns differ between antiestrogen-sensitive and -resistant breast cancer cells, with aggregates containing essential proteasome and UPR proteins found exclusively in endocrine-sensitive cells. Specifically, by inhibiting RTCB function, we were able to re-sensitize endocrine-resistant cells to tamoxifen treatment. Therefore, this work opens new avenues for research with the aim of studying the aggregated proteome as a novel approach to improve the response to antiestrogen therapy.

## 2. Materials and Methods

### 2.1. Cell Culture

MDA-MB-231 cells were grown in Dulbecco’s modified Eagle’s medium (DMEM) supplemented with 10% FBS and 5% PEST (Thermo Fisher Scientific, Bremen, Germany). MCF-7 and T-47D were grown in RPMI medium supplemented with 10% FBS and antibiotics. To obtain tamoxifen resistant cells (MCF-7R and T-47DR), endocrine responsive cells were grown in medium containing 500 nM 4-hydroxytamoxifen (TAM; Sigma-Aldrich, St. Louis, MO, USA) for 8 weeks, after which ER expression and cell number in response to TAM and ICI 182 780 (ICI; Sigma-Aldrich, St. Louis, MO, USA) were confirmed by cell counting and Western blot and showed to be either unaltered or increased (Supplementary Figure S1). This phenotype was tested every time the cells were thawed. To rule out clonal effects, MCF-7 and MCF-7R cell lines obtained from Dr. Julia Gee at Cardiff University were also used. MCF-7R and T-47DR cells were routinely grown in RPMI medium supplemented with 10% FBS, 100 nM TAM and antibiotics. All cell lines were grown at 37 °C in a humidified 5% CO2/95% air atmosphere. Twenty-four hours before treatment, the growth medium was replaced by phenol red–free RPMI 1640 or DMEM with 5% charcoal-treated FBS (DCC), 1% PEST and supplemented with 4 mM glutamine. ER ligands were dissolved in 100% ethanol (ETOH) and used at a final test concentration of 10 nM E2, 250 nM ICI, or 500 nM TAM. Autophagy inhibitors Bafilomycin (lysosomal inhibitor) or Spautin-1 (USP10 and USP13 inhibitor) were used at a final concentration of 1µM alone or in combination with 500 nM tamoxifen (TAM) for 8 h. MDA-MB-231, T-47D and MCF-7 cell lines were purchased from ATCC and used straight away, all cell lines were routinely checked for mycoplasma infection using PCR.

### 2.2. Cell Proliferation and Transfection Assays

The cells were plated in 24-well culture plates (5 × 10^4^ cells/mL) in phenol red-free medium with 5% DCC. On day 1 after plating, cells were treated with the different study conditions in triplicate. On day 2 and day 3, medium was renewed, and on day 5 each well was treated with trypsin and cells were counted using a Neubauer chamber. For small interfering RNA (siRNA)-mediated knockdown of RTCB, cells were transfected with 1 nM of either the targeting siRNA (RTCBi) or a control (RTCBc) sequence (Sigma-Aldrich) using HiPerFect (Qiagen, Hilden, Germany) or Lipofectamine RNAiMAX (Invitrogen, Carlsbad, CA, USA) for 24 h. For cell proliferation assays, cells were treated with TAM [500 nM] or equivalent volume of solvent (ETOH) for 72 h. For Western-blot hybridization or CASP-3 activity experiments cells were treated with 4µ8C (25 µM), Thapsigargin (Acros Organic, Geel, Belgium) (0.5 µM), TAM (500 nM) or equivalent volume of solvent (ETOH) for 3 h and 24 h, respectively.

### 2.3. Insoluble Protein Isolation

Cells were harvested in ELB lysis buffer (0.5% Triton-X100; 5% HEPES 1 M pH 7; 5% NaCl 5 M; 0.1% DTT 1 M; 0.1% NaF 1 M; 1% EGTA 0.1 M pH8; 0.4% EDTA 0.5 M; 1% Na3VO4 (all from Sigma-Aldrich); 2% Protease inhibitor 50× (Roche, Mannheim, Germany); 2.5% PMSF (Thermo Fisher Scientific, Bremen, Germany); H2O miliQ), and kept on ice while total protein was measured using a standard BSA assay. For insoluble protein fraction isolation 100 µg of total protein were diluted in 100 µL ELB buffer and centrifuged at 16,000 G for 20 min, 4 °C. The supernatant was discarded, and the pellet was resuspended in 80 µL ELB buffer +20 µL NP40 (Thermo Fisher Scientific). After sonication, the solution was centrifuged at 16,000 G for 20 min, 4 °C. The supernatant was discarded. For SDS-Page electrophoresis, the pellet was resuspended in 50 µL ELB buffer, and for Western blot hybridization the pellet was resuspended in 20 µL ELB buffer and a sonication step was performed. The total protein and insoluble protein extracts were stored at −80 °C until use.

### 2.4. SDS-PAGE Electrophoresis and Western Blot Hybridization

Proteins were denatured for 5 min at 95 °C in 6× SDS loading buffer (375 mM Tris-HCl; 9% SDS (Sigma Aldrich); 50% Glycerol; 0.03% Bromophenol blue (Thermo Fisher Scientific)). Twenty µg of total protein or 25 µL of insoluble protein were resolved by 7.5%, 10% or 12% SDS-PAGE. Thereafter, the bands were either visualized with Coomassie blue or transferred onto nitrocellulose membranes (Amersham, Darmstadt, Germany). Membranes were blocked with a solution of 5% BSA in 0.05% Tween-TBS and incubated overnight with the following primary antibodies: anti-ERα (sc-373863) 1:300, β-amyloid (sc-28365) 1:500, SQSTM1 (sc-28359) 1:1000, LC3 α/β (sc-398822) 1:800, XBP1 (sc-8015) 1:500, IRE1α (sc-390960) 1:500, PSMC2 (sc-166972) 1:500, PSMD11 (sc-517422) 1:500, PDIA6 (sc-365260) 1:500, all from Santa Cruz Biotechnology; RTCB (HPA000535, Sigma-Aldrich); Ubiquitin (GT7S1) 1:1000, GeneTex (Irvine, CA, USA); BIP (#3183) 1:800, PERK (#5683) 1:800, p-PERK (#3179) 1:800, Eif2α (#5324) 1:800 and p- Eif2α (#3597) 1:800, all from Cell Signalling Technology (Danvers, MA, USA). Immunoreactive products were visualized by chemiluminescence with a Bio-Rad ChemiDoc™ Imager or fluorescence with a LI-COR Odyssey 9120 Digital Imaging System. Protein loading was visualized by Ponceau staining. Band intensity quantification was performed by densitometry using the ImageLab digital densitometry software freely available for download from Biorad. For Western blots, band intensity was normalized using the Ponceau intensity of the whole lane. XBP-1s/XBP-1u ratio was calculated by dividing the intensity of both bands in the same membrane followed by ponceau normalization of the lane. Densitometry values of total lysates were first standardized using ponceau staining intensity and then used to normalize the insoluble protein levels. Intensity was compared between treatments of the same cell line. The graphs show relative changes compared to the untreated control in MCF-7 or T-47D cells.

### 2.5. Aggresome Detection and Co-Localization Studies

ProteoStat^®^ Aggresome Detection Kit Assay (Enzo Life Sciences, Farmingdale, NY, USA) was used for detection of protein aggregates in cells and tissues. ProteoStat^®^ is a 488 nm excitable fluorescent molecular probe which is like Thioflavin-T, a rotor molecule-dye that is essentially nonfluorescent until it binds to structural features associated with aggregated proteins [27]. All procedures using the ProteoStat^®^ Aggresome Detection Kit were conducted according to the manufacturer’s instructions using a positive control for protein aggregation that consisted of the same cells treated with the proteasome inhibitor MG132 run in parallel. Briefly, after cell fixation and permeabilization, unspecific dye binding was blocked with 10% FBS, 0.5% Triton X-100 in PBS for 1 h. Quantification of # of cells with protein aggregates (PA)/total # of cells was carried out in 3 independent experiments in which 6 to 8 random field images at 200× magnification were taken of each treatment (at least 10,000 cells per group were counted). Fluorescence microscopy was carried out in a Zeiss Axio Imager Z1 microscope (Zeiss, Oberkochen, Germany) equipped with a CCD monochromatic digital camera (Axiocam HRm) or in an Olympus IX-81 microscope (Olympus, Tokyo, Japan) equipped with a CCD monochromatic digital camera (F-view II). ImageJ digital open source software was used to calculate the numbers of positive stained cells and to measure the parameter correlated fluorescence [CF = fluorescence intensity—(area x mean fluorescence intensity of background]. For co-localization studies, ProteoStat^®^ [1:1000] was incubated for 30 min at room temperature (RT) with CellEventTMCaspase-3/7 Green (Invitrogen, Carlsbad, CA, USA), according to manufacturer’s instructions, or with the following antibodies: SQSTM1 (sc-28359, Santa Cruz Biotechnology) 1:500, LC3 α/β (sc-398822, Santa Cruz Biotechnology) 1:500, β-amyloid 1:400 or RTCB 1:200, incubated overnight at 4 °C. Fluorescence confocal microscopy was carried out in a Zeiss LSM-510 META confocal microscope (Zeiss, Oberkochen, Germany) or in a Zeiss LSM 880 confocal microscope with Airyscan.

### 2.6. Tryptic Digestion, Mass Spectrometry Analysis and Protein Identification

Tryptic digestion was performed according to [28], with a few modifications. Protein bands were manually excised from the gel and transferred to 1.5 mL plastic tubes. The gel pieces were washed one time with 25 mM ammonium bicarbonate, three times with 25 mM ammonium bicarbonate/50% acetonitrile (ACN, VWR Chemicals, Radnor, PA, USA) and one time with ACN. The protein’s cysteine residues were reduced with 10 mM DTT (45 min at 56 °C) and alkylated with 55 mM iodo-acetamide (30 min at RT). The gel pieces were washed with 25 mM ammonium bicarbonate, followed by 25 mM ammonium bicarbonate/50% ACN and finally with ACN. Gel pieces were dried in a SpeedVac (Savant, Thermo Fisher Scientific, Bremen, Germany) and rehydrated in digestion buffer containing 12.5 µg/mL sequencing grade modified trypsin (AbSCIEX) in 50 mM ammonium bicarbonate. After 30 min at 37 °C, the supernatant was removed and discarded, 50 μL of 50 mM ammonium bicarbonate were added and the samples were incubated overnight (for 16 h) at 37 °C. Extraction of tryptic peptides was performed by the addition of 5% formic acid (FA, Fluka, Mexico City, MX, USA) one time and 5% FA/50% CAN, twice. Tryptic peptides were lyophilized in a SpeedVac (Savant, Thermo Fisher Scientific, Bremen, Germany) and resuspended in 1% FA solution. The samples were analyzed with a Q Exactive Hybrid Quadrupole-Orbitrap (Thermo Fisher Scientific, Bremen, Germany) through the EASY-spray nano ESI source (Thermo Fisher Scientific, Bremen, Germany) that was coupled to an Ultimate 3000 (Dionex, Sunnyvale, CA, USA) nano HPLC (high-pressure liquid chromatography) system. The trap (5 mm × 300 µm I.d.) and the EASY-spray analytical (150 mm × 75 µm) columns used were C18 Pepmap100 (Dionex, LC Packings) with a particle size of 3 µm. Peptides were trapped at 30 μL/min in 96% solvent A (0.1% FA). Elution was achieved with the solvent B (0.1% formic acid/80% acetonitrile *v*/*v*) at 300 nL/min. The 92 min gradient used was as follows: 0–3 min, 4% solvent B; 3–70 min, 4–25% solvent B; 70–90 min, 25–40% solvent B; 90–92 min, 40–90% solvent B; 92–100 min, 90% solvent B; 100–101 min, 90–4% B, 101–120 min, 4% solvent B. The mass spectrometer was operated at 2.2 kV in the data dependent acquisition mode. A MS2 method was used with a FT survey scan from 400 to 1600 *m*/*z* (resolution 70,000; AGC target 1E6). The 10 most intense peaks were subjected to HCD fragmentation (resolution 17,500; AGC target 5E4, NCE 28%, max. injection time 100 ms, dynamic exclusion 35 s). Spectra were processed and analyzed using Proteome Discoverer (version 2.2, Thermo Fisher Scientific, Bremen, Germany), with the MS Amanda (version 2.0, University of Applied Sciences Upper Austria, Research Institute of Molecular Pathology, Vienna, Austria) and Sequest HT search engines. Uniprot (TrEMBL and Swiss-Prot) protein sequence database (version of May 2017) was used for all searches under Homo Sapiens. Database search parameters were as follows: carbamidomethylation of cysteine, oxidation of methionine, and the allowance for up to two missed tryptic cleavages. The peptide mass tolerance was 10 ppm, and fragment ion mass tolerance was 0.02 Da. To achieve a 1% false discovery rate, the Percolator (version 2.0, Thermo Fisher Scientific, Bremen, Germany) node was implemented for a decoy database search strategy, peptides were filtered for high confidence and a minimum length of 6 amino acids and proteins were filtered for a minimum number of 1 peptide sequence. Only proteins with mean values above 25% vs. control (ETOH) condition were considered as upregulated in the insoluble fraction.

### 2.7. Statistical Analysis and Bioinformatics

Statistical analysis was performed using GraphPad Prism software version 6.0. Data from at least two independent experiments were used to calculate the mean ± standard deviation (SD). Mann-Whitney tests were used for the comparison of two independent groups for immunofluorescence, Western blotting, cell proliferation and siRNA assays. Wilcoxon tests were used for analysis of human breast cancer paired tissue samples. Differences were considered significant if *p* < 0.05. Venny 2.1 [29] was used to identify the upregulated proteins uniquely found in MCF-7 cells treated with TAM or ICI. For this, first up-regulated proteins in each treatment were obtained by comparison to the control (ETOH), second the upregulated proteins by E2 were deleted from the list, and finally up-regulated proteins by the antiestrogens in MCF-7 were compared to those found in MCF-7R cells.

### 2.8. Patient Sample Collection and Characterization

The clinicopathological data of the patient cohort are presented in Supplementary Table S1. The samples used in this study are within the project “Exploring epigenetic profiling as prognostic/predictive markers of endocrine resistance in estrogen receptor positive breast cancer” approved by IPO Porto’s Ethical Committee (CES IPO: 369/2017).

## 3. Results

### 3.1. Aggresome Accumulation Correlates with Reduced UPR and Autophagy Activation in Antiestrogen-Sensitive Breast Cancer Cells

**Figure 1:**
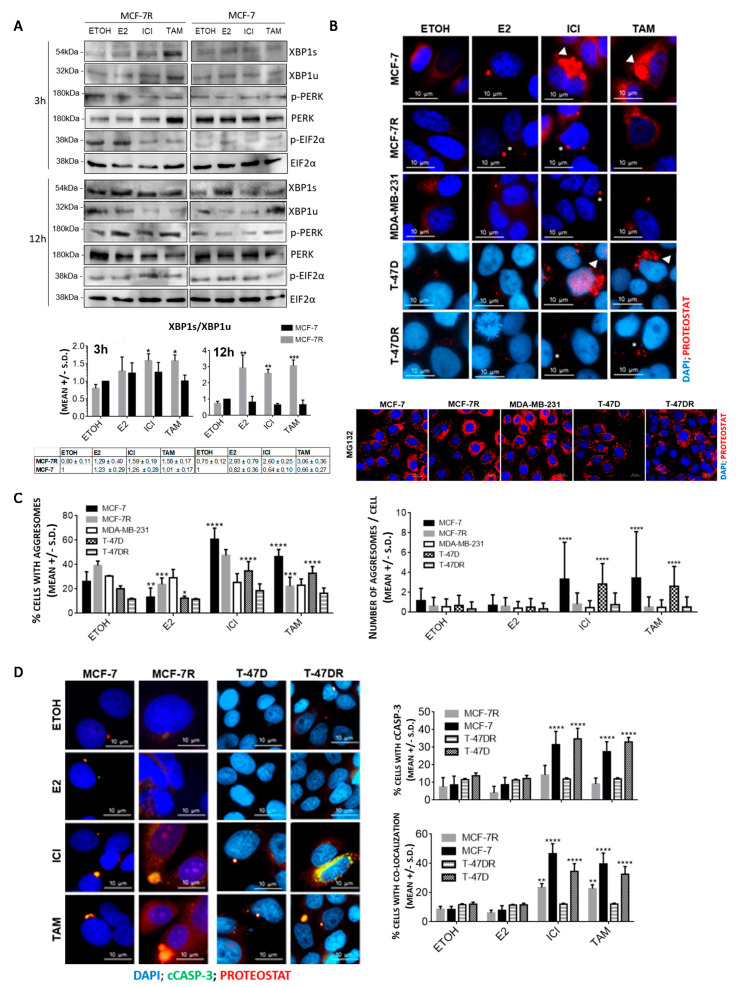
Characterization of antiestrogen-induced protein aggregation clearance and accumulation in breast cancer cell lines. (**A**) Western blot showing XBP1s/XBP1u ratio and activation of PERK/EIF2α UPR branch after exposure to 10 nM 17β-estradiol (E2), 250 nM Fulvestrant (ICI) or 500 nM 40HO-tamoxifen (TAM). * *p* < 0.05; ** *p* < 0.01; *** *p* < 0.001 vs. ETOH in the same cell line. Blots are representative of two experiments. (**B**) Comparative analysis of protein aggregation levels in MCF-7, MCF-7R, MDA-MB-231, T-47D and T-47DR cell lines. Staining with Proteostat^®^ was carried out after 24 h treatment with 10 nM E2, 250 nM ICI, 500 nM TAM or 5 μM MG132 (positive control for protein aggregation). Arrowheads: cytoplasmatic localization; star: juxtanuclear region. (**C**) The graphs summarize results from at least three independent experiments, as shown in (**D**), with a total of 10,000 cells counted. **** *p* < 0.0001, *** *p* < 0.001, ** *p* < 0.01; vs. same cell line ETOH. D. Proteostat^®^ and cleaved caspase-3 (cCASP-3) were colocalized by a double ICC after 24 h incubation with 10 nM E2, 250 nM ICI, 500 nM TAM. **** *p* < 0.0001, ** *p* < 0.01 vs. same cell line ETOH. Representative of at least two experiments. In all cases, the cells received the same concentration of solvent (absolute ETOH).

In antiestrogen-resistant (MCF-7R) cells, the XBP-1s/XBP-1u ratio increased already after 3 h incubation with TAM or ICI and increased throughout the 12 h evaluated, while in MCF-7 cells it did not significantly change (Figure 1A). We ruled out the possibility that MCF-7 cells could be partially compensating EnR stress by activation of the PERK/EIF2A pathway, since neither the ratio p-PERK/PERK nor p-EIF2A/EIF2A increased following TAM or ICI treatment (Figure 1A). To exclude the possibility that E2 could influence the effect of the antiestrogens alone, we carried out a control experiment co-incubating the cells with TAM + E2 and observed the same effects as with TAM alone (Supplementary Figure S3A). Therefore, the model of antiestogen resistance used herein shows efficient activation of the IRE1α/XBP1 branch in response to antiestrogens, in line with other studies [4,6].

The lack of UPR activation by MCF-7 cells in response to antiestrogens alone or combined with E2 resulted in protein aggregation and a significant increase in the aggresome levels as well as the % of cells containing aggresomes (Figure 1B,C and Supplementary Figure S3B). Once again, co-incubation of E2 + TAM produced the same effect as TAM alone. This was also observed in T-47D cells, but not in MCF-7R or T-47DR cells. Treatment with E2, and antiestrogenic treatment in cells which do not express ERα (MDA-MB-231 cells) did not produce aggresome accumulation (Figure 1B,C). This indicates that aggresomes result from antagonizing ERα function in cells that are sensitive to ICI and TAM. The aggresomes were found mostly dispersed in the cytoplasm of sensitive cells, indicating that the protein quality control (PQC) mechanisms were saturated [30], but in MCF-7R and T47-DR cells they were preferably found at the juxtanuclear quality control region that concentrates proteasomes and disaggregating chaperones [31,32], suggesting a functional PQC (Figure 1B,C and Supplementary Figure S3B,C, arrowheads).

Aggresomes are preferably cleared by autophagy [33]; differences in autophagic patterns between MCF-7 and MCF-7R cells were evident already in basal conditions and were maintained after exposure to antiestrogens, with MCF-7R cells showing clear p62 and LC3-II puncta colocalization with aggresomes (Supplementary Figure S4A). On the other hand, in MCF-7 the number of puncta was lower, with a more diffuse p62 and LC3-II staining and higher number of aggresomes that did not colocalize (Supplementary Figure S4A). These results are compatible with lower autophagic efficiency in antiestrogen sensitive cells as compared to resistant ones. This is in agreement with the literature [7,34] and the lack of increase in XBP-1s [4], as shown in Figure 1A. It has previously been shown that cyto-protective autophagy can contribute to antiestrogen resistance [7,8,22,35,36], and several groups have shown that inhibition of autophagy can re-sensitize ERα+ cells to antiestrogen treatment [7,8,9,22,34,37,38,39]. Moreover, co-incubation of MCF-7R cells with TAM and the autophagy inhibitors bafilomycin or spautin-1 for 24 h increased the aggresome load and their co-localization with LC-3 or p62 (Supplementary Figure S4B), supporting the notion that enhanced autophagy protects the cells from aggresome accumulation. Aggresome-associated cell death results from toxic gain of function by facilitating aberrant protein–protein interactions [40], or loss of function of essential proteins [41]. Sensitive and resistant cells showed nearly complete aggresomes colocalization with cleaved caspase 3 (cCASP-3) after antiestrogen treatment (not shown); consequently, the number of cells positive for aggresome/cCASP-3 colocalization was significantly higher in antiestrogen-treated MCF-7 and T-47D cells (Figure 1D).

In summary, resistant cells are more capable of clearing protein aggregates that result from antiestrogen treatment because they maintain XBP1s expression and have more efficient autophagy; while in sensitive cells, the lack of aggresome clearance correlates with antiestrogen-induced cell death.

### 3.2. Protein Aggregation after Antiestrogen Treatment Targets Different Pathways in Sensitive and Resistant Cells

**Figure 2:**
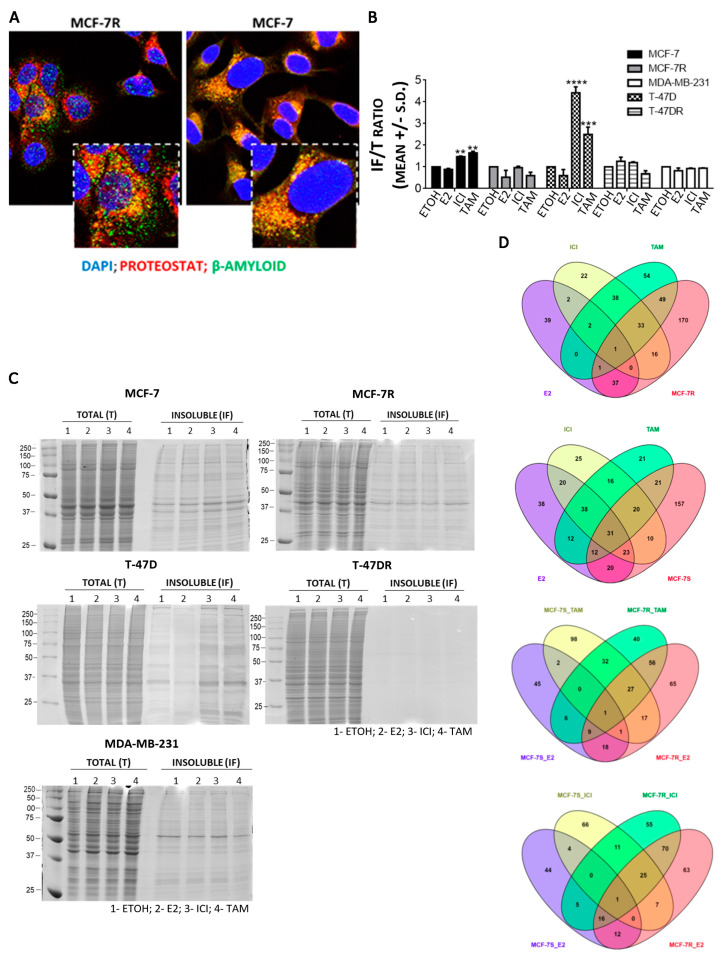
Characterization of aggregated proteins in breast cancer cells. (**A**) Confocal images of cells treated with 5 μM MG132 for 24 h and co-stained with anti-Amyloid A4/β-amyloid domain antibody and Proteostat^®^. (**B**,**C**) SDS-PAGE resolution of total (T) and insoluble fractions (IFs) of antiestrogen-sensitive and resistant cells treated for 24 h with ETOH (control), 10 nM E2, 250 nM ICI, 500 nM TAM. **** *p* < 0.0001, *** *p* < 0.001, ** *p* < 0.01 vs. same cell line ETOH, non-parametric *t* test. (**D**) Venn diagrams showing the distribution of proteins identified by MS/MS as upregulated (>30% vs. ETOH) in the insoluble fraction of MCF-7 cells vs. the insoluble induced by E2, TAM or ICI in MCF-7R (top) and MCF-7R (bottom) vs. the insoluble induced by E2, TAM or ICI in MCF-7 cells.

To isolate protein aggregates for subsequent mass spectrometry analysis, we enriched in proteins that were insoluble in ionic detergent. These included intrinsically disordered proteins, like Amyloid A4 proteins and β-amyloids, which, using MG132, accumulate in Proteostat-stained aggresomes (Figure 2A). The detergent-insoluble and total protein fraction were separated by SDS-PAGE and exhibited an increased insoluble/total protein ratio (IF/T) only in antiestrogen-sensitive cells after treatment (Figure 2B,C). Notably, E2 protected proteins from detergent-induced destabilization, and the ERα-negative MDA-MB-231 cells did not exhibit increased levels of insoluble proteins (Figure 2B,C). The insoluble fractions of MCF-7 and MCF-7R cells were analyzed by LC-MS/MS to identify the pathways that could be hindered by loss of protein function due to aggregation. A total of 640 and 605 proteins were consistently found in the detergent-insoluble fraction of MCF-7 and MCF-7R cells, respectively. Treatment of MCF-7 cells with TAM or ICI for 24 h resulted in 38 proteins aggregated with TAM or ICI, 54 with TAM treatment and 22 with ICI (Figure 2D, top panel, and Table 1). For comparison, the unique proteins aggregated in MCF-7R cells after TAM or ICI treatment were also analyzed (Figure 2D, second from top, and Supplementary Table S2).

**Figure 3:**
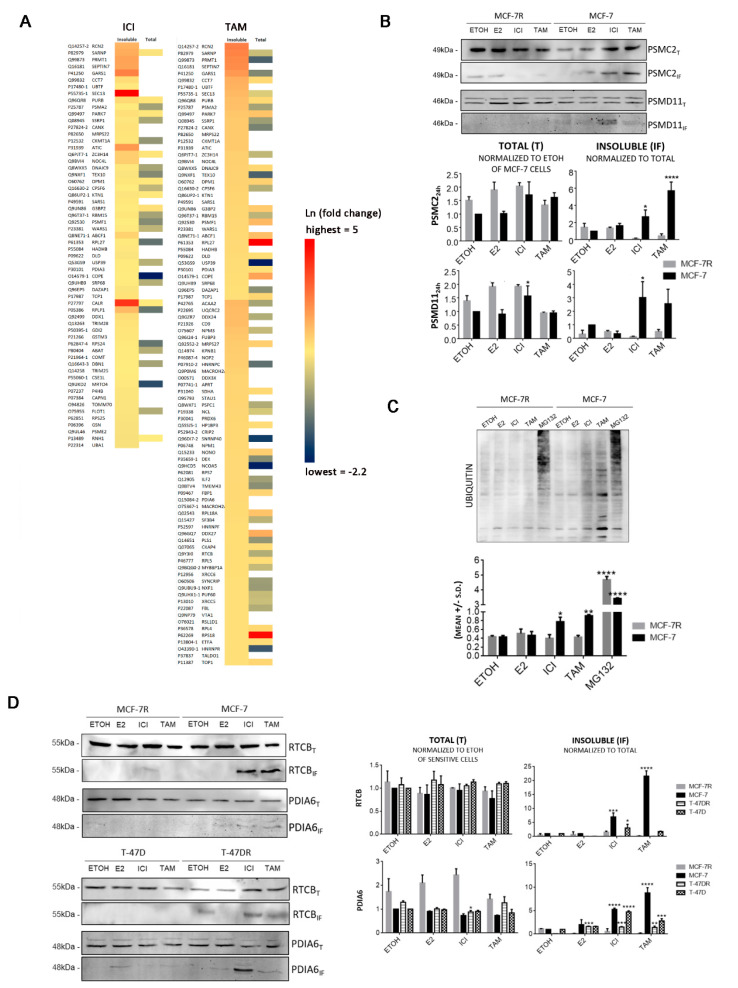
Protein aggregation induced by antiestrogens in MCF-7 cells. Cells were treated with 10 nM E2, 250 nM ICI or 500 nM TAM or the same volume of ETOH (control) for 24 h. Total (T) and detergent-insoluble (IF) protein fractions were analyzed by LC-MS/MS or Western blot. (**A**) Heat map showing fold-change differences (cutoff 30%) identified by LC-MS/MS. White spaces are proteins that were present in the insoluble fraction but not detected in the total protein fraction. (**B**) Western blot of total and insoluble PSMD11 and PSMC2. (**C**) Western blot showing total ubiquitin levels; 5 µM MG132 was used as positive control. (**D**) Western blot of RTCB and PDIA6 and band intensity quantification. In all cases, results are representative of two experiments. **** *p* < 0.0001, *** *p* < 0.001, ** *p* < 0.01 and * *p* < 0.05 vs. ETOH in the same cell line, non-parametric *t* test.

The aggregated proteins in MCF-7 cells significantly represented Proteasome pathway (pTAM = 0.003 and pICI = 7.03x10^−05^; Table 2). Additionally, TAM aggregated mRNA surveillance proteins (*p* = 0.015), while ICI targeted Protein processing in endoplasmic reticulum (*p* = 0.027). These pathways were not overrepresented by the detergent-insoluble proteins from MCF-7R after either TAM or ICI treatment. Instead, MCF-7R cells possessed detergent-insoluble proteins enriched in Huntington’s disease (pICI = 0.002). We also identified proteins with relevance in proteinopathies in MCF-7 cells, including Poly(U)-binding-splicing factor PUF60 (PUF60), associated with amyotrophic lateral sclerosis (ALS) [42,43,44], Transaldolase-1 (TALDO1), associated with Parkinson’s disease [45], and hydroxyacyl-CoA dehydrogenase trifunctional multienzyme complex subunit beta (HADHB), related to Creutzfeldt-Jakob disease [46]. To rule out higher levels of detergent-insoluble proteins being a result of higher expression levels, we compared the 640 proteins found to be insoluble in MCF-7 cells with their values in the total protein extract and found no significant correlation (Spearman rTAM = 0.019 and rICI = 0.13). Additionally, when analyzed individually, the total levels of the proteins found to be uniquely aggregated in MCF-7 cell after antiestrogen treatment were not up-regulated in the total protein fraction (Figure 3A).

### 3.3. Protein Aggregation after Antiestrogen Treatment Impairs Proteasome and UPR Function

Proteosome pathway proteins that consistently aggregated after TAM and ICI treatment where PSMC5 and PSMC4 in either MCF-7 or MCF-7R cells. PSMA2 and UBA1 were also only detected as aggregated in MCF-7 cells with either of the antiestrogens, with PSMD11 and PSMC2 mostly aggregated after ICI treatment (Table 1 and Supplementary Table S2). We confirmed using Western blot that even when aggregated in both sensitive and resistant cells lines, PSMD11 and PSMC2 were aggregated in higher levels in MCF-7 cells (Figure 3B). Total levels of ubiquitinated proteins increased in MCF-7 cells after TAM or ICI treatment (Figure 3C), which strongly supports the notion that in sensitive cells, antiestrogens impair the proteasome function by inducing aggregation of several 26S proteasome subunits.

RTCB and PDIA6 were also uniquely aggregated in MCF-7 cells (Figure 3D). RTCB catalyzes the ligation of XBP-1 mRNA following cleavage by IRE-1α endonuclease [47], while PDIA6 maintains IRE-1α activation within a physiologically acceptable range [48]. Total RTCB and PDIA6 did not change, although the insoluble proteins significantly increased only in MCF-7 and T-47D cells (Figure 3D). These results reinforce the idea that although RTCB and PDIA6 are expressed at similar levels in both sensitive and resistant cell lines, they are more prone to aggregation in sensitive cells in response to ERα antagonism, which may lead to cytotoxicity due to their loss of function. In support of this idea, RTCB aggregation occurred already after 3h (Supplementary Figure S5A,B) and correlated with lack of XBP1s protein increase only in sensitive cells (Figure 1A). Moreover, RTCB subcellular localization is related to its dual function as t-RNA ligase (nuclear) [49] and XBP1 ligase (cytoplasm). We confirmed that in sensitive cells treated with antiestrogens, RTCB is initially localized in nuclear speckles but also in aggresomes, and after 24 h, RTCB was mostly found in the aggresomes, while in resistant cells, RTCB did not colocalize with aggresomes (Supplementary Figure S5B). E2 treatment showed a clear RTCB distribution throughout the cytoplasm and nucleus of MCF-7 cells. As a control, we combined E2+TAM and evaluated the pattern of RTCB staining in relation to the aggresomes after 24 h, where it was clear that addition of E2 did not prevent TAM-mediated RTCB aggregation (Supplementary Figure S5C). Therefore, the RTCB instability triggered by antiestrogens in MCF-7 cells, impairs XBP1s-mediated proteostasis, promoting a vicious cycle of protein misfolding and aggresome accumulation.

### 3.4. Inhibition of RTCB Expression Downregulates XBP1s and Improves Sensitivity to Tamoxifen

**Figure 4:**
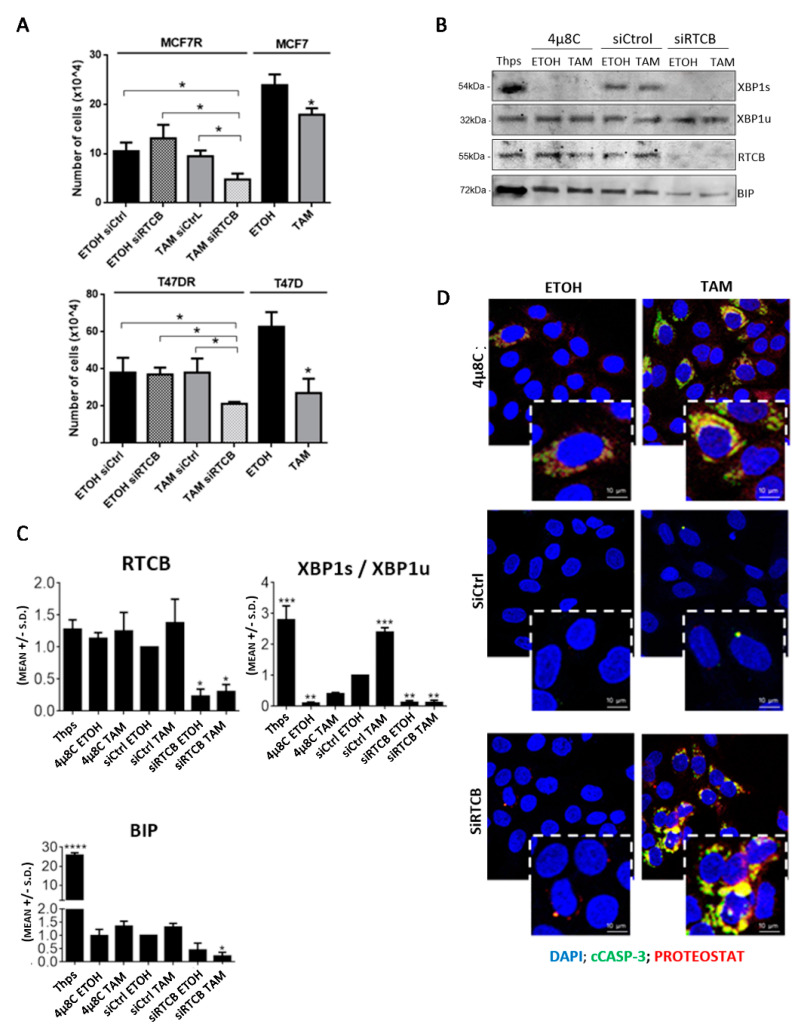
Inhibition of RTCB expression with siRNAs restores sensitivity to TAM in antiestrogen-resistant cell lines. (**A**) Knock-down of RTCB with siRNAs in MCF-7R and T-47DR cells for 24 h prior treatment with 500 nM TAM + siRNAs for 3 days. Comparisons for the same cell line vs. other treatments, * *p* < 0.05. Experiments are representative of three. (**B**) The effect of RTCB knock-down on XBP1s, XBP1u and BiP protein levels in MCF-7R cells was assessed and compared side-by-side to the effect of the IRE1α activator Thapsigargin (Thps; 0.5 µM) and inhibitor 4µ8C (25 µM). (**C**) Quantification of results shown in (**B**). Comparisons vs. siCtrl ETOH ***: *p* < 0.001, **: *p* < 0.01; *: *p* < 0.05, **** *p* < 0.0001, non-parametric *t* test. (**D**) Aggresome and cleaved CASP3 (cCASP-3) colocalization in cells treated for 24 h with 500nM TAM in combination with siRNA for RTCB or 25 µM 4µ8C.

To test whether RTCB function promotes survival to antiestrogens, we pre-treated MCF-7R cells with siRNAs to RTCB (siRTCB) for 24 h, after which they were treated with TAM + siRTCB for an additional 3 days. siRTCB significantly decreased proliferation of TAM-treated cells (Figure 4A). The reversal of resistance to TAM was as efficient as the inhibition of proliferation observed in MCF-7 cells and was confirmed in T-47D/T-47DR cells. Moreover, similar to the IRE1α inhibitor 4µ8C, RTCB reduced the XBP1s/XBP1u ratio (Figure 4B,C). In contrast to 4µ8C, siRTCB downregulated BIP protein (Figure 4B,C), suggesting that blocking RTCB function may translate into longer lasting inhibition of UPR possibly due to the weak stability of the IRE1α-4µ8C complex [50]. Blocking XBP1s production by either siRTCB or 4µ8C in MCF-7R treated with TAM resulted in increased aggresome formation and co-localization with cCASP-3 (Figure 4D). Interestingly, in siRTCB-treated cells, XBP1s and BIP levels were also reduced with ETOH, but protein aggregation was only observed after challenging the cells with TAM, confirming that RTCB function supports resistance to TAM.

**Figure 5:**
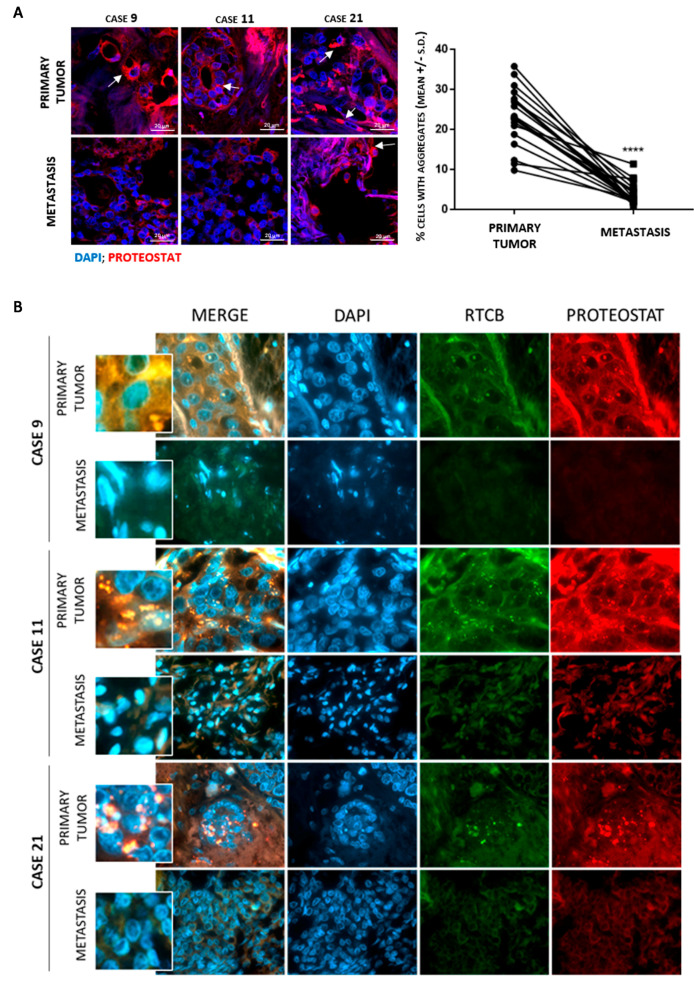
Protein aggregation levels and RTCB aggregation in luminal breast cancer. Human primary breast adenocarcinomas that were therapy naïve with their paired metastasis arising after completion of endocrine-therapy were analyzed by confocal microscopy. (**A**) Protein aggregation detected as aggresome accumulation using Proteostat^®^. ****: *p* < 0.0001; Wilcoxon test for paired samples. (**B**) Representative images of Proteostat^®^ and RTCB co-localization.

To investigate the significance of RTCB aggregation in vivo, we tested a small cohort consisting of 22 paired samples of untreated primary tumor and the matched metastasis that appeared after completion of endocrine treatment (Supplementary Table S1). A statistically significant decrease in the percentage of cells with protein aggregates was found in metastatic tumors that represent disease recurrence after completion of endocrine treatment (Figure 5A). Aggregation of RTCB showed higher colocalization with aggresomes in the primary tumor as compared to the metastasis (Figure 5B). This supports the idea that protein aggregation in breast cancer tissue samples may provide information about tumor sensitivity to endocrine therapy and suggest that antiestrogen resistant cells are better capable of preventing RTCB aggregation.

## 4. Discussion

Enhancement of UPR and autophagy have been linked to endocrine therapy resistance [5,22]. Our results go a step further, showing that antiestrogen treatment causes less aggresome accumulation in resistant cells due to maintenance of soluble/functional RTCB, needed to maintain the protective effect of IRE1α/XBP1 pathway. Loss of RTCB function leads to aggresome accumulation, which can be used to predict antiestrogen response.

To date, the association of higher XBP1s with antiestrogen resistance had been related to co-activation of ERα by XBP-1s [51]. Here, we show that RTCB aggregation induced by antiestrogens and accumulation in aggresomes occurs only in sensitive cells and correlated with reduced XBP1s and BIP levels. Therefore, RTCB function could be targeted to revert endocrine resistance by reducing ERα co-activation and protection from BIP chaperoning activity. We tested this hypothesis using RTCB knock-down in antiestrogen-resistant cells to demonstrate that RTCB loss of function reproduced the aggresome pattern of sensitive cells and re-sensitized to tamoxifen. Notably, RTCB knock-down produced a much more stable and powerful inhibition of the stress response mediated by XBP1s than the IRE1α inhibitor 4μ8C, (measured as BiP protein levels). This may be because unfavorable pharmacokinetics limit the utility of 4μ8C and other aldehydes that function as IRE1α inhibitors [50]. Thus, RTCB targeting can potentially inhibit this pathway in a more stable manner.

Aggregated beta-amyloids are commonly found in proteinopathies [40] and have been associated with toxic gain of function through aberrant protein–protein interactions or loss of function [43,52]. Protein aggregates are known to remain insoluble in strong detergent buffers so, biochemical fractionation methods that involve the sequential use of buffers and detergents of increasing stringency and ultracentrifugation to separate the soluble and insoluble fractions have been applied to tissue and cellular homogenates [53,54,55]. The multistep protocol used in this study (Supplementary Figure S2) allows, at first, the separation of cytosolic soluble proteins and in a second phase, NP40 allows the solubilization and separation of membrane proteins. Additionally, as internal control, we were able to identify several proteins previously reported to be insoluble such as cytoskeletal proteins, collagens and matrix proteins [53] or with increased tendency to aggregate like TALDO1 [45], HADHB [46], TAR DNA-binding protein 43 (TDP-43) or PUF60 [42,43,44]. Interestingly, the number of aggregated proteins identified in the detergent-insoluble fraction of MCF-7 and MCF-7R cells was similar, although the identity and levels of specific proteins varied between the two phenotypes and did not correlate with total protein levels. This clearly shows that antiestrogens induce the aggregation of specific proteins. It has recently been shown that protein solubility increases when cells are undergoing cell division due to post-translational modifications regulating enzymatic activity [56]. In future studies it will be interesting to identify the stimuli that triggers this selective aggregation or protection of proteins.

Differential aggresome formation was found in therapy naïve primary luminal breast cancer vs. their metastasis, appearing after endocrine therapy. Only a small cohort was analyzed; however, the results are promising, as the metastases may have developed strategies to prevent RTCB aggregation. Future studies of luminal primary tumors from women that were treated with endocrine treatment, in which some developed early resistance/progression and others did not are needed. It is noteworthy that resistance to antiestrogens has been linked to emergence or selection of cancer stem-like cells [57,58]. There is an increasing body of evidence that points to important roles of UPR and autophagy in cancer stem-like subpopulaitons. Specifically, impairment of autophagy in breast cancer stem cells reduces expression of staminal markers, self-renewal capacity and tumorigenicity [59,60,61]. Autophagy is upregulated in mammospheres where Beclin1 and ATG4 are needed to maintain and expand the breas cancer stem-like populations [59,61,62]. UPR modulation in breast cancer stem-like cells has been less explored, yet it has been shown that BIP is enriched in these subpopulations isolated from multiple breast cancer cell lines [63] and activation of the IRE1/XBP1 branch was related to the maintenance breast cance stem cells, their tumorigenicity and resistance to chemotherapy [64,65]. Studies in other models have shown that damaged proteins are assymetrically partitioned during cell division [66,67,68]. Therefore, in the future, it will be interesting to explore whether protein aggregation load could function as a driving force for cancer stem-like features, through asymmetric partitioning and may explain the lack of protein aggregation in the metastasis arising after endocrine therapy.

**Figure 6:**
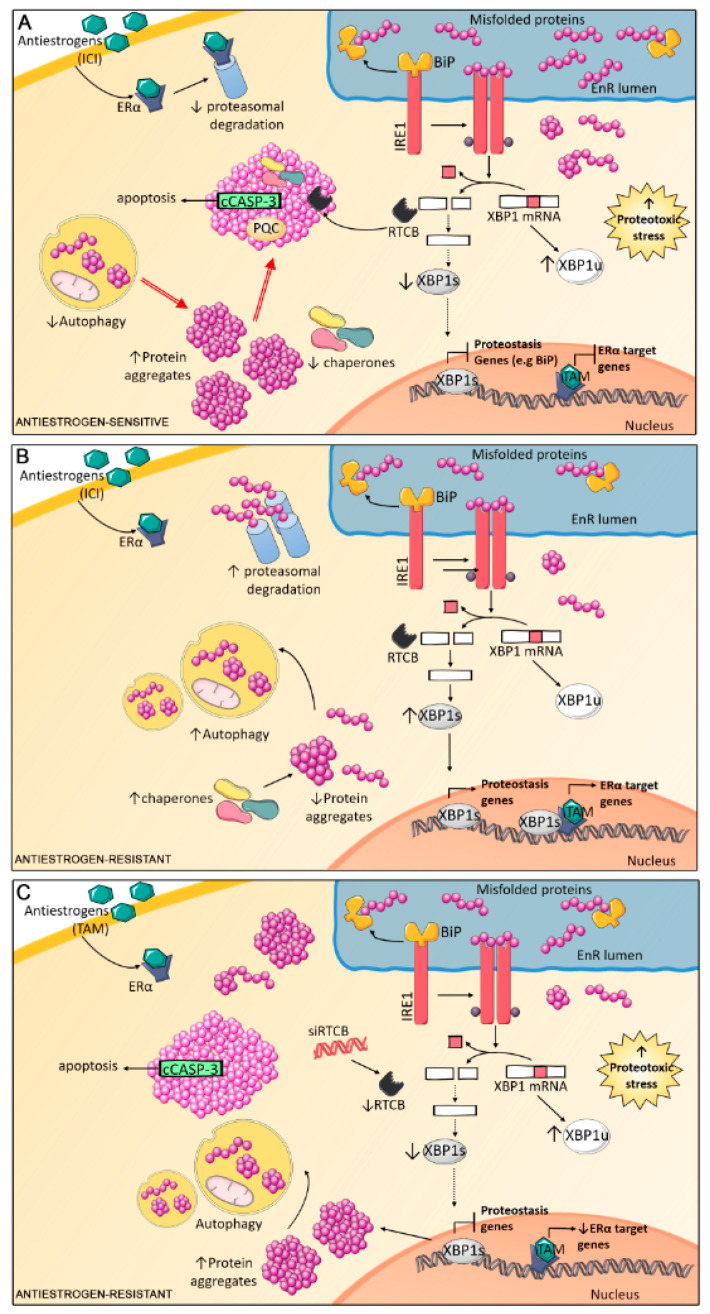
Schematic representation of the findings presented in this work in the context of previously described mechanisms guiding endocrine resistance. (**A**) In antiestrogen-sensitive cells, treatment with antiestrogens induces ERα proteasomal degradation (ICI) or inhibits the transcription of ERα target genes (TAM); consequently, proliferation and survival signaling is halted. Additionally, antiestrogen therapy increases oxidative and metabolic stress, which hinders correct protein folding to increase their aggregation. The results presented herein show that the PQC network (autophagy, UPR and proteasome) may be rapidly saturated leading to aggresome accumulation. Aggresomes contain essential proteins needed to overcome proteotoxic stress (i.e., RTCB, chaperones and PQC proteins), reducing their function. Aggregated RTCB cannot complete XBP1 splicing, XBP1 levels drop and, consequently, the activation of proteostasis genes under XBP1 regulation, like BiP, becomes compromised. Altogether, these events may generate a positive feedback loop increasing the accumulation of aggresomes which, in turn, potentiates the proteome instability and contributes to impair stress responses. Activation of pro-apoptotic pathways with co-localization of cleaved caspase 3 (cCASP-3) in aggresomes ultimately results in cellular death. (**B**) Antiestrogen-resistant cells have a higher autophagic and proteasomal degradation capacity, which, coupled with induced expression of XBP1s and transactivation of its target genes (i.e., BiP, chaperones, ERAD response) allows resistant cells to cope with protein misfolding and aggregation, keeping aggresome levels low. In addition, co-activation of ERα by XBP1s is possibly maintained, resulting in increased proliferation and treatment with antiestrogens do not compromise survival signaling. (**C**) RTCB knock-down in resistant cells treated with antiestrogens, results in impaired XBP1 expression which is sufficient to promote aggresome accumulation. Impaired transcription of proteostasis genes by XBP1s possibly contributes to saturate the PQC network. Intolerable proteome instability and consequent proteotoxic stress may activate pro-apoptotic pathways resulting in cellular death as observed by cCASP3 accumulation. EnR—endoplasmic reticulum; Era—estrogen receptor alpha; PQC—protein quality control proteins.

In this work, we presented evidence concerning a new approach to identify antiestrogen response and disclosed RTCB as a novel player in controlling acquired antiestrogen resistance (Figure 6). Cell stress induced by antiestrogen treatment (i.e., induced by ROS or metabolic imbalances) [69,70] results in protein misfolding. Since antiestrogen-sensitive cells have lower UPR and autophagic capacity [7,34], decreased proteasomal activity [71] and expression of chaperones [35,72], and the protein quality control mechanisms become saturated and aggresomes accumulate. Proteins trapped into aggresomes are essential to overcome proteotoxic stress (i.e., RTCB, proteasome proteins and chaperones) and their loss of function leads to a disruptive cycle of proteostasis loss leading to activation of apoptosis mediated by cCASP3 translocation to aggresomes. This is illustrated by the analysis of RTCB, which does not complete XBP1 splicing (dotted lines), and therefore, transcription of XBP1s target genes (i.e., BiP) is not activated. These alterations enhance EnR stress and may also decrease activation of ERα-mediated proliferation by XBP1s [73]. On the other hand, proliferation and survival signaling remains active in resistant cells treated with antiestrogens. Since these cells have increased activation of their proteostasis mechanisms, misfolded proteins are easily cleared and since XBP1s are induced upon antiestrogen treatment, aggresome levels are kept low. In addition, co-activation of ERα by XBP1s may be possible, sustaining proliferation and survival. However, if RTCB is knocked-down in resistant-cells treated with antiestrogens, reduced levels of XBP1s are sufficient to promote aggresome accumulation and apoptosis.

## 5. Conclusions

Taken together, the findings presented herein suggest that protein aggregation patterns could be used to predict resistance to antiestrogen therapy, and they could be indicative of improved capacity to maintain a healthy proteome. This work opens a new avenue for research with respect to finding breast cancer prognostic markers and therapeutic targets, where the identification of proteins prone to aggregate could help identify antiestrogen response and understand mechanisms of disease.

## Figures and Tables

**Table 1:** Proteins found to be uniquely aggregated in MCF-7 cells following 24 h incubation with 4OH-tamoxifen (TAM) or Fulvestrant (ICI).

Uniprot IDs	Entry Name	Protein Name	Gene Names
		TAM	
P42765	THIM_HUMAN	3-ketoacyl-CoA thiolase, mitochondrial	ACAA2
P22695	QCR2_HUMAN	Cytochrome b-c1 complex subunit 2, mitochondrial	UQCRC2
Q9GZR7	DDX24_HUMAN	ATP-dependent RNA helicase DDX24	DDX24
P21926	CD9_HUMAN	CD9 antigen	CD9 MIC3 TSPAN29 GIG2
O75607	NPM3_HUMAN	Nucleoplasmin-3	NPM3
Q96I24-1	FUBP3_HUMAN	Far upstream element-binding protein 3	FUBP3 FBP3
Q92552-2	RT27_HUMAN	28S ribosomal protein S27, mitochondrial	MRPS27 KIAA0264
Q14974	IMB1_HUMAN	Importin subunit beta-1	KPNB1 NTF97
P46087-4	NOP2_HUMAN	Probable 28S rRNA	NOP2 NOL1 NSUN1
P07910-2	HNRPC_HUMAN	Heterogeneous nuclear ribonucleoproteins C1/C2	HNRNPC HNRPC
Q9P0M6	H2AW_HUMAN	Core histone macro-H2A.2	MACROH2A2 H2AFY2
O00571	DDX3X_HUMAN	ATP-dependent RNA helicase DDX3X	DDX3X DBX DDX3
P07741-1	APT_HUMAN	Adenine phosphoribosyltransferase	APRT
P31040	SDHA_HUMAN	Succinate dehydrogenase [ubiquinone] flavoprotein subunit, mitochondrial	SDHA SDH2 SDHF
O95793	STAU1_HUMAN	Double-stranded RNA-binding protein Staufen homolog 1	STAU1 STAU
Q8WXF1	PSPC1_HUMAN	Paraspeckle component 1	PSPC1 PSP1
P19338	NUCL_HUMAN	Nucleolin	NCL
P30041	PRDX6_HUMAN	Peroxiredoxin-6	PRDX6 AOP2 KIAA0106
Q5SSJ5-1	HP1B3_HUMAN	Heterochromatin protein 1-binding protein 3	HP1BP3
P52943-2	CRIP2_HUMAN	Cysteine-rich protein 2	CRIP2 CRP2
Q96DI7-2	SNR40_HUMAN	U5 small nuclear ribonucleoprotein 40 kDa protein	SNRNP40 PRP8BP SFP38
P06748	NPM_HUMAN	Nucleophosmin	NPM1 NPM
Q15233	NONO_HUMAN	Non-POU domain-containing octamer-binding protein	NONO NRB54
P35659-1	DEK_HUMAN	Protein DEK	DEK
Q9HCD5	NCOA5_HUMAN	Nuclear receptor coactivator 5	NCOA5 KIAA1637
P62081	RS7_HUMAN	40S ribosomal protein S7	RPS7
Q12905	ILF2_HUMAN	Interleukin enhancer-binding factor 2	ILF2 NF45 PRO3063
Q9BTV4	TMM43_HUMAN	Transmembrane protein 43	TMEM43 UNQ2564/PRO6244
P09467	F16P1_HUMAN	Fructose-1,6-bisphosphatase 1	FBP1 FBP
Q15084-2	PDIA6_HUMAN	Protein disulfide-isomerase A6	PDIA6 ERP5 P5 TXNDC7
O75367-1	H2AY_HUMAN	Core histone macro-H2A.1	MACROH2A1 H2AFY
Q02543	RL18A_HUMAN	60S ribosomal protein L18a	RPL18A
Q15427	SF3B4_HUMAN	Splicing factor 3B subunit 4	SF3B4 SAP49
P52597	HNRPF_HUMAN	Heterogeneous nuclear ribonucleoprotein F	HNRNPF HNRPF
Q96GQ7	DDX27_HUMAN	Probable ATP-dependent RNA helicase DDX27	DDX27 cPERP-F RHLP
Q14651	PLSI_HUMAN	Plastin-1	PLS1
Q07065	CKAP4_HUMAN	Cytoskeleton-associated protein 4	CKAP4
Q9Y3I0	RTCB_HUMAN	RNA-splicing ligase RtcB homolog	RTCB C22orf28 HSPC117
P46777	RL5_HUMAN	60S ribosomal protein L5	RPL5 MSTP030
Q9BQG0-2	MBB1A_HUMAN	Myb-binding protein 1A	MYBBP1A P160
P12956	XRCC6_HUMAN	X-ray repair cross-complementing protein 6	XRCC6 G22P1
O60506	HNRPQ_HUMAN	Heterogeneous nuclear ribonucleoprotein Q	SYNCRIP HNRPQ NSAP1
Q9UBU9-1	NXF1_HUMAN	Nuclear RNA export factor 1	NXF1 TAP
Q9UHX1-1	PUF60_HUMAN	Poly(U)-binding-splicing factor PUF60	PUF60 FIR ROBPI SIAHBP1
P13010	XRCC5_HUMAN	X-ray repair cross-complementing protein 5	XRCC5 G22P2
P22087	FBRL_HUMAN	rRNA 2’-O-methyltransferase fibrillarin	FBL FIB1 FLRN
Q9NP79	VTA1_HUMAN	Vacuolar protein sorting-associated protein VTA1 homolog	VTA1 C6orf55 HSPC228 My012
O76021	RL1D1_HUMAN	Ribosomal L1 domain-containing protein 1	RSL1D1 CATX11 CSIG PBK1
P36578	RL4_HUMAN	60S ribosomal protein L4	RPL4 RPL1
P62269	RS18_HUMAN	40S ribosomal protein S18	RPS18 D6S218E
P13804-1	ETFA_HUMAN	Electron transfer flavoprotein subunit alpha, mitochondrial	ETFA
O43390-1	HNRPR_HUMAN	Heterogeneous nuclear ribonucleoprotein R	HNRNPR HNRPR
P37837	TALDO_HUMAN	Transaldolase	TALDO1 TAL TALDO TALDOR
P11387	TOP1_HUMAN	DNA topoisomerase 1	TOP1
		ICI	
P27797	CALR_HUMAN	Calreticulin	CALR CRTC
P05386	RLA1_HUMAN	60S acidic ribosomal protein P1	RPLP1 RRP1
Q92499	DDX1_HUMAN	ATP-dependent RNA helicase DDX1	DDX1
Q13263	TIF1B_HUMAN	Transcription intermediary factor 1-beta	TRIM28 KAP1 RNF96 TIF1B
P50395-1	GDIB_HUMAN	Rab GDP dissociation inhibitor beta	GDI2 RABGDIB
P21266	GSTM3_HUMAN	Glutathione S-transferase Mu 3	GSTM3 GST5
P62847-4	RS24_HUMAN	40S ribosomal protein S24	RPS24
P80404	GABT_HUMAN	4-aminobutyrate aminotransferase, mitochondrial	ABAT GABAT
P21964-1	COMT_HUMAN	Catechol O-methyltransferase	COMT
Q16643-3	DREB_HUMAN	Drebrin	DBN1 D0S117E
Q14258	TRI25_HUMAN	E3 ubiquitin/ISG15 ligase TRIM25	TRIM25 EFP RNF147 ZNF147
P55060-1	XPO2_HUMAN	Exportin-2	CSE1L CAS XPO2
Q9UKD2	MRT4_HUMAN	mRNA turnover protein 4 homolog	MRTO4 C1orf33 MRT4
P07237	PDIA1_HUMAN	Protein disulfide-isomerase	P4HB ERBA2L PDI PDIA1
P07384	CAN1_HUMAN	Calpain-1 catalytic subunit	CAPN1 CANPL1 PIG30
O94826	TOM70_HUMAN	Mitochondrial import receptor subunit TOM70	TOMM70 KIAA0719 TOM70
O75955	FLOT1_HUMAN	Flotillin-1	FLOT1
P62851	RS25_HUMAN	40S ribosomal protein S25	RPS25
P06396	GELS_HUMAN	Gelsolin	GSN
Q9UL46	PSME2_HUMAN	Proteasome activator complex subunit 2	PSME2
P13489	RINI_HUMAN	Ribonuclease inhibitor	RNH1 PRI RNH
P22314	UBA1_HUMAN	Ubiquitin-like modifier-activating enzyme 1	UBA1 A1S9T UBE1
		TAM or ICI	
Q14257-2	RCN2_HUMAN	Reticulocalbin-2	RCN2 ERC55
P82979	SARNP_HUMAN	SAP domain-containing ribonucleoprotein	SARNP HCC1 HSPC316
Q99873	ANM1_HUMAN	Protein arginine N-methyltransferase 1	PRMT1 HMT2 HRMT1L2 IR1B4
Q16181	SEPT7_HUMAN	Septin-7	SEPTIN7 CDC10 SEPT7
P41250	GARS_HUMAN	Glycine-tRNA ligase	GARS1 GARS
Q99832	TCPH_HUMAN	T-complex protein 1 subunit beta	CCT7 CCTH NIP7-1
P17480-1	UBF1_HUMAN	Nucleolar transcription factor 1	UBTF UBF UBF1
P55735-1	SEC13_HUMAN	Protein SEC13 homolog	SEC13 D3S1231E SEC13A
Q96QR8	PURB_HUMAN	Transcriptional activator protein Pur-beta	PURB
P25787	PSA2_HUMAN	Proteasome subunit alpha type-2	PSMA2 HC3 PSC3
Q99497	PARK7_HUMAN	Parkinson disease protein 7	PARK7
Q08945	SSRP1_HUMAN	FACT complex subunit SSRP1	SSRP1 FACT80
P27824-2	CALX_HUMAN	Calnexin	CANX
P82650	RT22_HUMAN	28S ribosomal protein S22, mitochondrial	MRPS22 C3orf5 RPMS22
P12532	KCRU_HUMAN	Creatine kinase U-type, mitochondrial	CKMT1A CKMT; CKMT1B
P31939	PUR9_HUMAN	Bifunctional purine biosynthesis protein ATIC	ATIC PURH OK/SW-cl.86
Q6PJT7-1	ZC3HE_HUMAN	Zinc finger CCCH domain-containing protein 14	ZC3H14
Q9BVI4	NOC4L_HUMAN	Nucleolar complex protein 4 homolog	NOC4L
Q8WXX5	DNJC9_HUMAN	DnaJ homolog subfamily C member 9	DNAJC9
Q9NXF1	TEX10_HUMAN	Testis-expressed protein 10	TEX10 L18 Nbla10363
O60762	DPM1_HUMAN	Dolichol-phosphate mannosyltransferase subunit 1	DPM1
Q16630-2	CPSF6_HUMAN	Cleavage and polyadenylation specificity factor subunit 6	CPSF6 CFIM68
Q86UP2-1	KTN1_HUMAN	Kinectin	KTN1 CG1 KIAA0004
P49591	SYSC_HUMAN	Serine-tRNA ligase, cytoplasmic	SARS1 SARS SERS
Q9UN86	G3BP2_HUMAN	Ras GTPase-activating protein-binding protein 2	G3BP2 KIAA0660
Q96T37-1	RBM15_HUMAN	RNA-binding protein 15	RBM15 OTT OTT1
Q92530	PSMF1_HUMAN	Proteasome inhibitor PI31 subunit	PSMF1
P23381	SYWC_HUMAN	Tryptophan--tRNA ligase, cytoplasmic	WARS1 IFI53 WARS WRS
Q8NE71-1	ABCF1_HUMAN	ATP-binding cassette sub-family F member 1	ABCF1 ABC50
P61353	RL27_HUMAN	60S ribosomal protein L27	RPL27
P55084	ECHB_HUMAN	Trifunctional enzyme subunit beta, mitochondrial	HADHB MSTP029
P09622	DLDH_HUMAN	Dihydrolipoyl dehydrogenase, mitochondrial	DLD GCSL LAD PHE3
Q53GS9	SNUT2_HUMAN	U4/U6.U5 tri-snRNP-associated protein 2	USP39 CGI-21 HSPC332
P30101	PDIA3_HUMAN	Protein disulfide-isomerase A3	PDIA3 ERP57 ERP60 GRP58
O14579-1	COPE_HUMAN	Coatomer subunit epsilon	COPE
Q9UHB9	SRP68_HUMAN	Signal recognition particle subunit SRP68	SRP68
Q96EP5	DAZP1_HUMAN	DAZ-associated protein 1	DAZAP1
P17987	TCPA_HUMAN	T-complex protein 1 subunit alpha	TCP1 CCT1 CCTA

**Table 2:** Kegg pathways represented by the aggregated proteins uniquely identified as upregulated in MCF-7 and MCF-7R cell lines after 24 h treatment with 250 nM ICI or 500 nM TAM.

MCF-7	TAM		ICI	
**Term**	**# Proteins**	***p* Value**	**# Proteins**	***p* Value**
hsa03010:Ribosome	25	3.89 × 10^−19^	6	0.011
hsa03040:Spliceosome	23	5.88 × 10^−17^	11	6.71 × 10^−7^
hsa03015:mRNA surveillance pathway	6	0.015		
hsa03050:Proteasome	4	0.033	6	7.03 × 10^−5^
hsa03008:Ribosome biogenesis in eukaryotes	5	0.050		
hsa01130:Biosynthesis of antibiotics	8	0.053		
hsa03013:RNA transport	7	0.057		
hsa00020:Citrate cycle (TCA cycle)	3	0.083		
hsa00280:Valine, leucine and isoleucine degradation			4	0.011
hsa01130:Biosynthesis of antibiotics			7	0.019
hsa04141:Protein processing in endoplasmic reticulum			6	0.027
hsa04612:Antigen processing and presentation			4	0.040
**MCF-7R**	**TAM**		**ICI**	
**Term**	**# Proteins**	***p* Value**	**# Proteins**	***p* Value**
hsa03010:Ribosome	39	2.12 × 10^−39^	17	1.17 × 10^−10^
hsa03040:Spliceosome	22	3.91 × 10^−16^	29	2.10 × 10^−25^
hsa03008:Ribosome biogenesis in eukaryotes	5	0.045		
hsa05168:Herpes simplex infection	7	0.063	8	0.020
hsa05412:Arrhythmogenic right ventricular cardiomyopathy (ARVC)	4	0.084		
hsa05016:Huntington’s disease			10	0.002

## Data Availability

The data presented in this study are available in the article and supplementary material.

## Supplementary Materials

The following are available online at https://www.mdpi.com/article/10.3390/cancers13133195/s1, Figure S1. Estrogen receptor expression and response to antiestrogenic treatment of MCF-7, MCF-7R, T-47D, T-47DR and MDA-MB-231 cell lines, Figure S2: Schematic representation of the insoluble fraction recovery protocol used in this study, Figure S3: Characterization of antiestrogen-induced protein aggregation clearance and accumulation in breast cancer cell lines, Figure S4: Correlation between autophagy activation and aggresome accumulation following antiestrogen treatment, Figure S5: Changes in RTCB expression levels in the total (T) and Insoluble Fractions (IF) of MCF-7 and MCF-7R cells along a period of 24h following ETOH (control), 10 nM E2, 250 nM ICI or 500 nM TAM treatments, Table S1: Clinicopathological data of the patient cohort, Table S2: Proteins found uniquely aggregated in MCF-7R cells following 24 h incubation with 4OH-tamoxifen (TAM) or Fulvestrant (ICI). Full pictures of the Western blots are list in the supplementary materials.
