# Supplementary material for: Protein Aggregation Patterns Inform about Breast Cancer Response to Antiestrogens and Reveal the RNA Ligase RTCB as Mediator of Acquired Tamoxifen Resistance
Source: Cancers (Basel). 2021 Jun 26;13(13):3195. doi: 10.3390/cancers13133195 (PMC8269126; doi:10.3390/cancers13133195)

In Figure S1

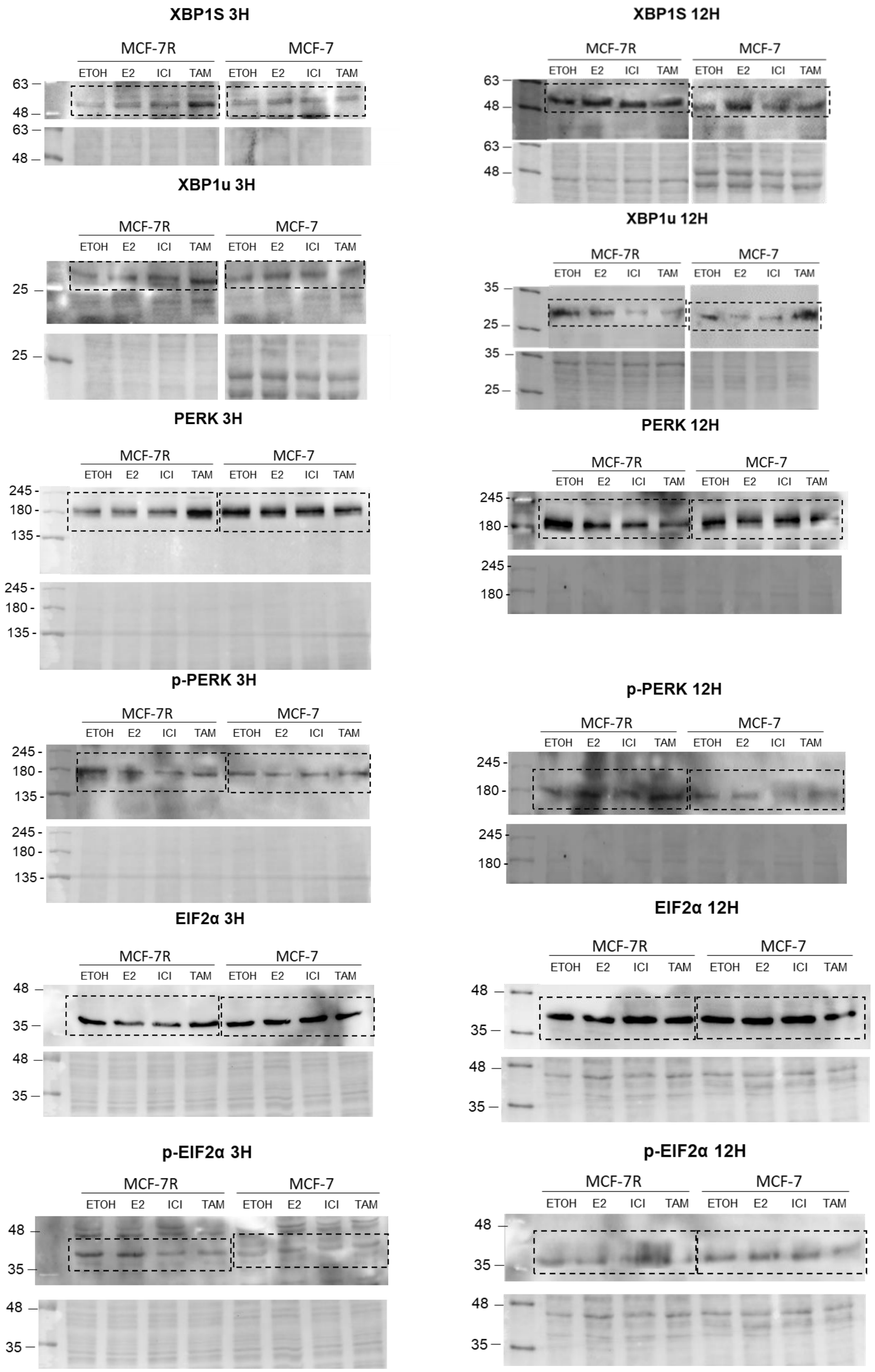
XBP1S 3H
MCF-7R
MCF-7
ETOH E2 ICI TAM ETOH E2 ICI TAM
63
48
63
48
XBP1S 12H
MCF-7R
MCF-7
ETOH E2 ICI TAM ETOH E2 ICI TAM
63
48
63
48
XBP1u 3H
MCF-7R
MCF-7
ETOH E2 ICI TAM ETOH E2 ICI TAM
25
25
XBP1u 12H
MCF-7R
MCF-7
ETOH E2 ICI TAM ETOH E2 ICI TAM
35
25
35
25
PERK 3H
MCF-7R
MCF-7
ETOH E2 ICI TAM ETOH E2 ICI TAM
245
180
135
245
180
135
PERK 12H
MCF-7R
MCF-7
ETOH E2 ICI TAM ETOH E2 ICI TAM
245
180
245
180
p-PERK 3H
MCF-7R
MCF-7
ETOH E2 ICI TAM ETOH E2 ICI TAM
245
180
135
245
180
135
p-PERK 12H
MCF-7R
MCF-7
ETOH E2 ICI TAM ETOH E2 ICI TAM
245
180
245
180
EIF2α 3H
MCF-7R
MCF-7
ETOH E2 ICI TAM ETOH E2 ICI TAM
48
35
48
35
EIF2α 12H
MCF-7R
MCF-7
ETOH E2 ICI TAM ETOH E2 ICI TAM
48
35
48
35
p-EIF2α 3H
MCF-7R
MCF-7
ETOH E2 ICI TAM ETOH E2 ICI TAM
48
35
48
35
p-EIF2α 12H
MCF-7R
MCF-7
ETOH E2 ICI TAM ETOH E2 ICI TAM
48
35
48
35

In Figure 3 B

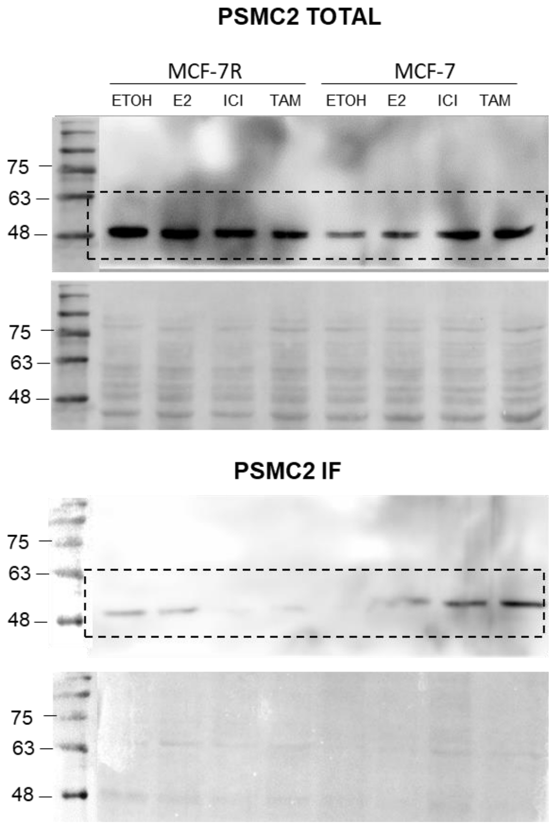

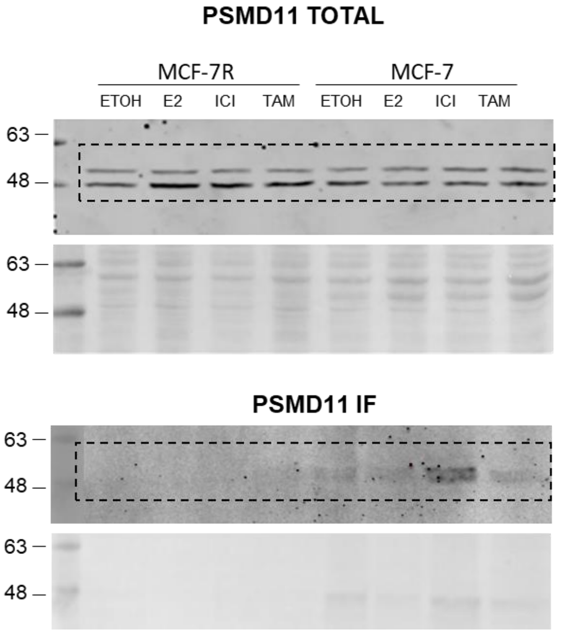

In Figure 3 C

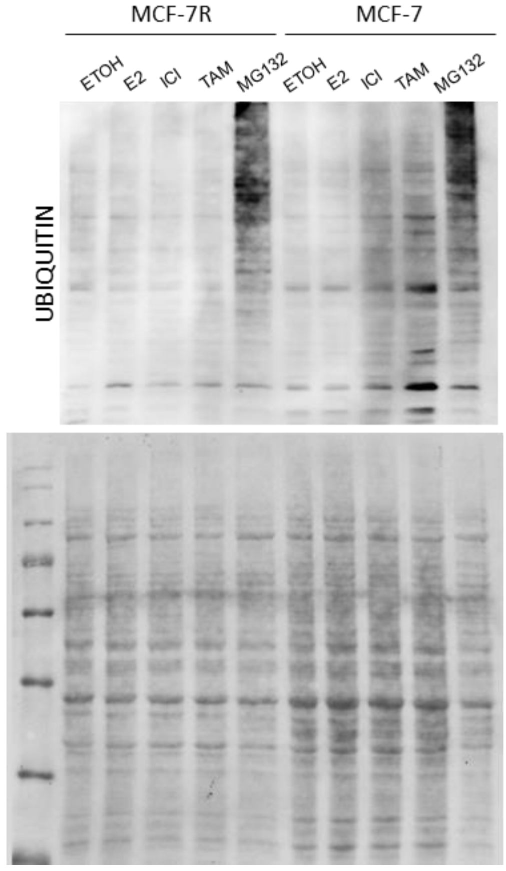

In Figure 3 D

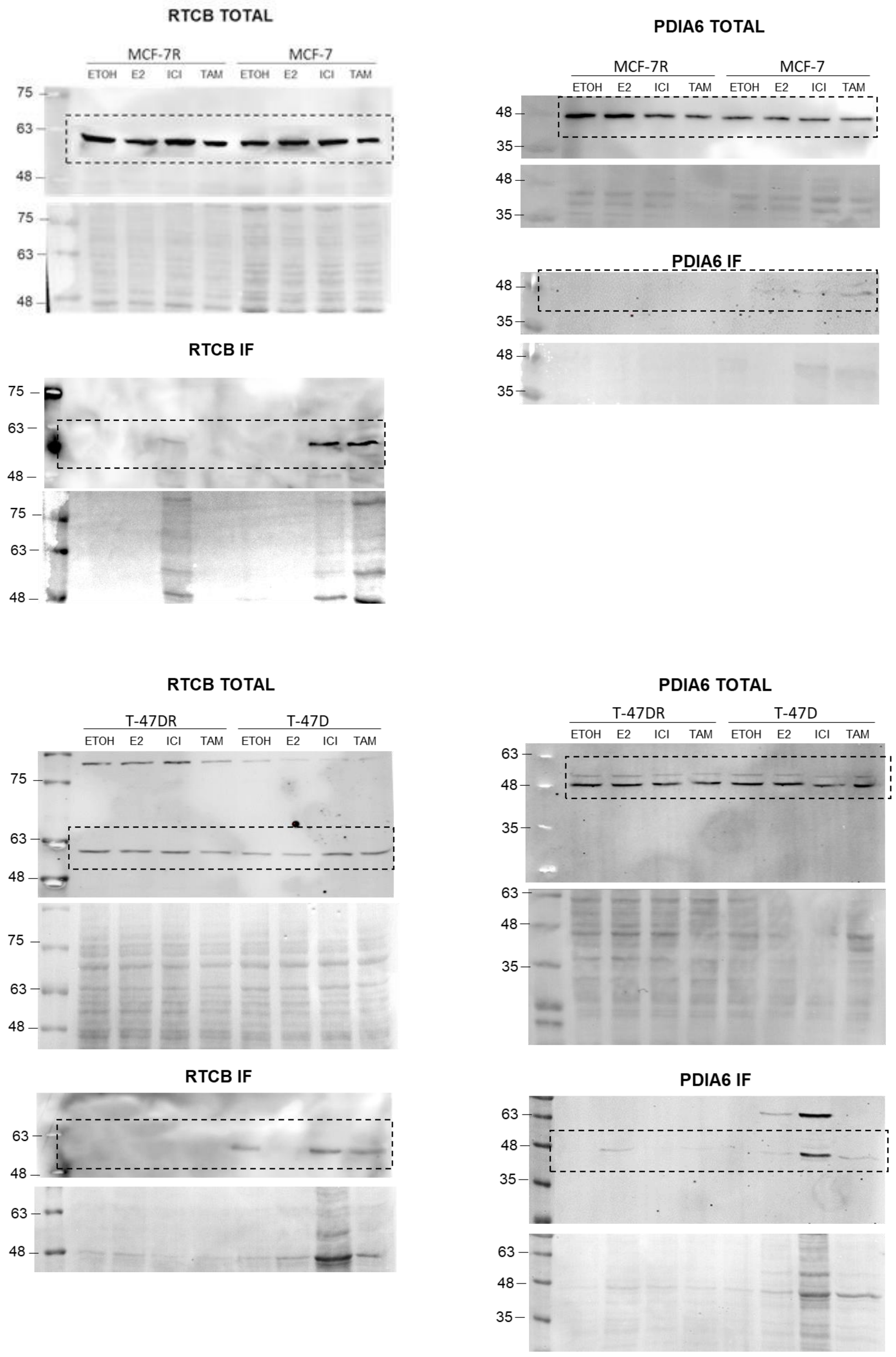
RTCB TOTAL
MCF-7R
MCF-7
ETOH E2 ICI TAM ETOH E2 ICI TAM
75
63
48
RTCB IF
PDIA6 TOTAL
PDIA6 IF
35
T-47DR
T-47D

In Figure 4B

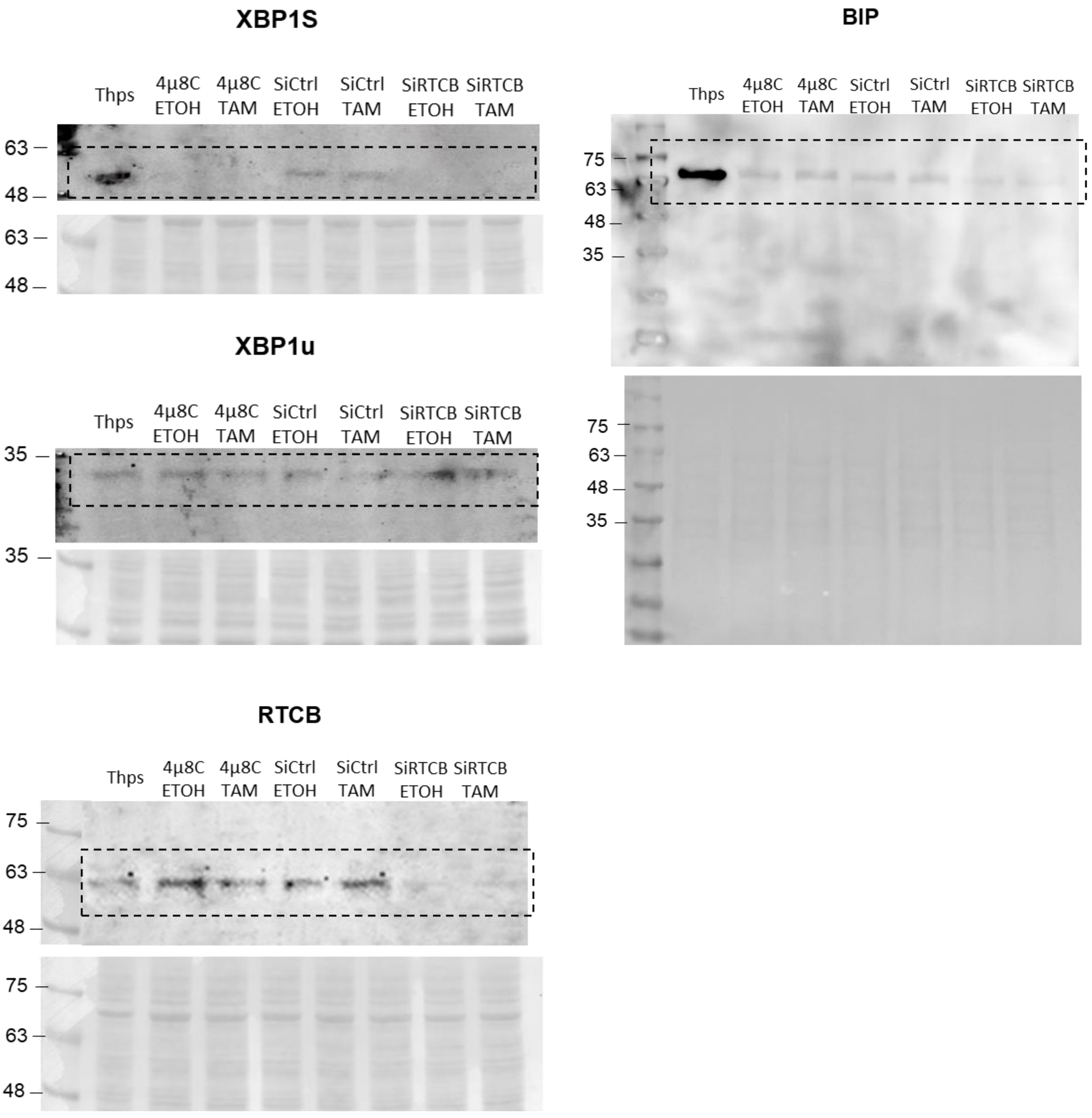
XBP1S
Thps
4µ8C ETOH
4µ8C TAM
SiCtrl ETOH
SiCtrl TAM
SiRTCB ETOH
SiRTCB TAM
63
48
63
48
XBP1u
Thps
4µ8C ETOH
4µ8C TAM
SiCtrl ETOH
SiCtrl TAM
SiRTCB ETOH
SiRTCB TAM
35
35
RTCB
Thps
4µ8C ETOH
4µ8C TAM
SiCtrl ETOH
SiCtrl TAM
SiRTCB ETOH
SiRTCB TAM
75
63
48
75
63
48
BIP
Thps
4µ8C ETOH
4µ8C TAM
SiCtrl ETOH
SiCtrl TAM
SiRTCB ETOH
SiRTCB TAM
75
63
48
35
75
63
48
35

In Figure S1

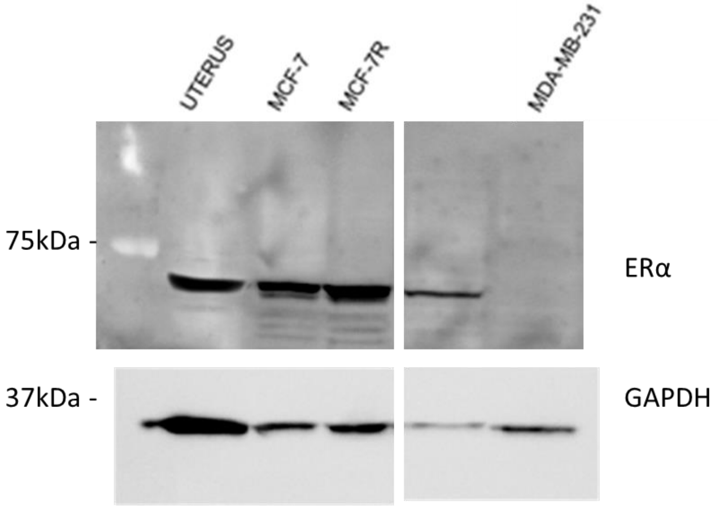
UTERUS
MCF-7
MCF-7R
MDA-MB-231
75kDa -
ERα
37kDa -
GAPDH

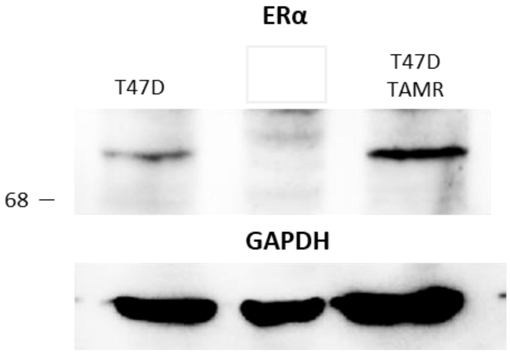
ERα
T47D
T47D
TAMR
68 —
GAPDH

In Figure 4

RTCB TOTAL 3H

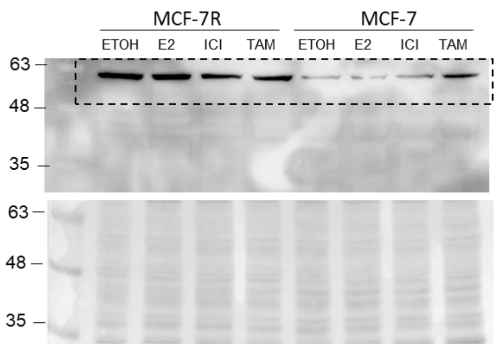

RTCB IF 3H

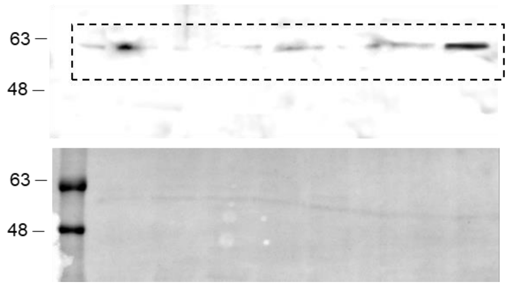

RTCB TOTAL 12H

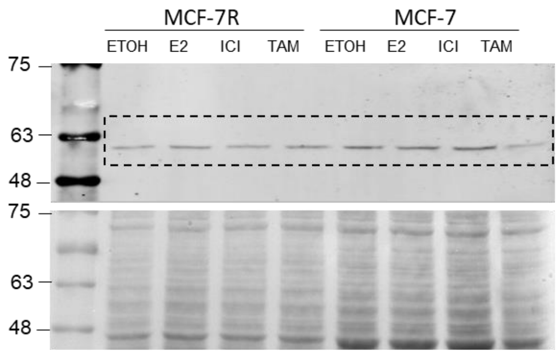

RTCB IF 12H

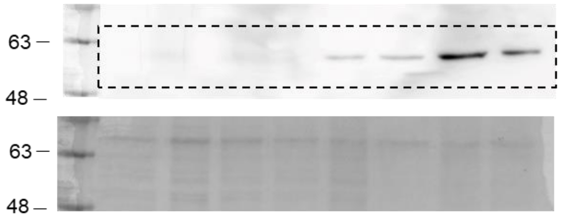

RTCB TOTAL 24H

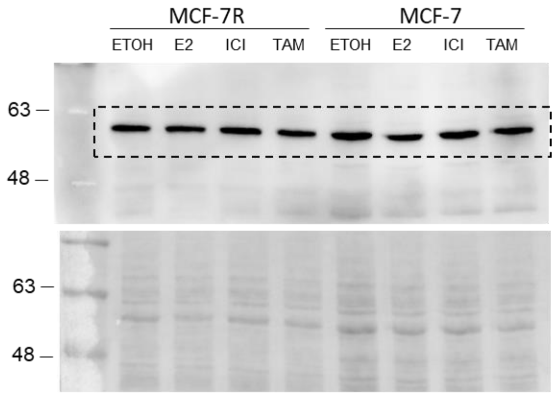

RTCB IF 24H

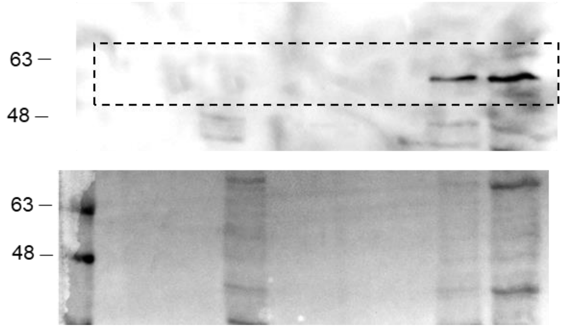

Supplement: Supplementary file 1 [file cancers-13-03195-s001.zip › cancers-1239423-supplementary/suppl/cancers-1239423-original-images.pdf]
