# Supplementary material for: Protein Aggregation Patterns Inform about Breast Cancer Response to Antiestrogens and Reveal the RNA Ligase RTCB as Mediator of Acquired Tamoxifen Resistance
Source: Cancers (Basel). 2021 Jun 26;13(13):3195. doi: 10.3390/cancers13133195 (PMC8269126; doi:10.3390/cancers13133195)
Supplement: Supplementary file 1 [file cancers-13-03195-s001.zip › cancers-1239423-supplementary/suppl/cancers-1239423-supplementary.pdf]

Supplementary files

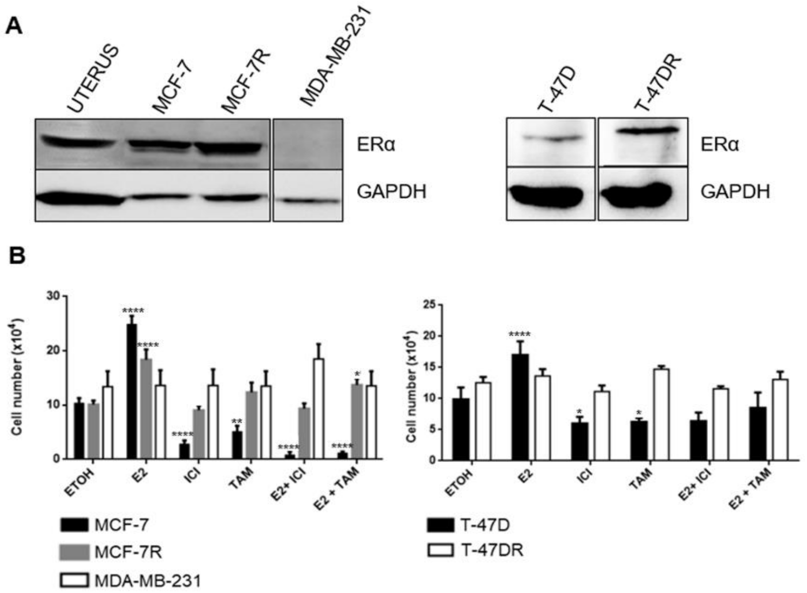

Figure S1. Estrogen receptor expression and response to antiestrogenic treatment of MCF-7, MCF-7R, T-47D, T-47DR and MDA-MB-231 cell lines. (**A**) Western blot showing ER*α* expression in different cell lines that were generated for this work. All the lanes presented correspond to samples separated in the same gel, the vertical lines indicate that the digital image was cut to show lanes side-by side. The blots are representative of two independent experiments. (**B**) Cell number evaluated following 5 days of treatment with 10 nM E2, 250 nM ICI, 500 nM TAM, 10 nM E2+250 nM ICI or 10 nM E2+500 nM TAM or the control consisting of same volume of ethanol (ETOH). ****$p < 0.0001$, ***$p < 0.001$, **$p < 0.01$ and *$p < 0.05$ vs. ETOH in the same cell line, non-parametric t test.

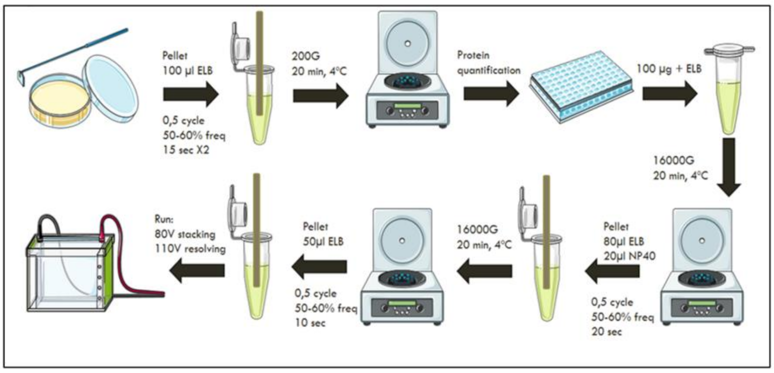

Figure S2. Schematic representation of the insoluble fraction recovery protocol used in this study. Cells were harvested in ELB lysis buffer, sonicated, centrifuged and kept on ice while total protein was measured using a standard BSA assay. For insoluble protein fraction isolation 100 µg of total protein were diluted in 100 µL ELB buffer and centrifuged at 16,000G for 20 min, 4 °C to separate cytosolic soluble proteins. The supernatant containing cytosolic soluble proteins was discarded, and the pellet was resuspended in 80 µL ELB buffer + 20 µL NP40. After sonication, the solution was centrifuged at 16,000G for 20 min, 4 °C to allow the solubilization and separation of membrane proteins. The supernatant was discarded. For SDS-Page electrophoresis the pellet enriched in detergent insoluble proteins was resuspended in 50 µL ELB buffer and for Western blot hybridization the pellet was resuspended in 20 µL ELB buffer and a sonication step was performed. The total protein and insoluble protein extracts were stored at -80 ºC until use.

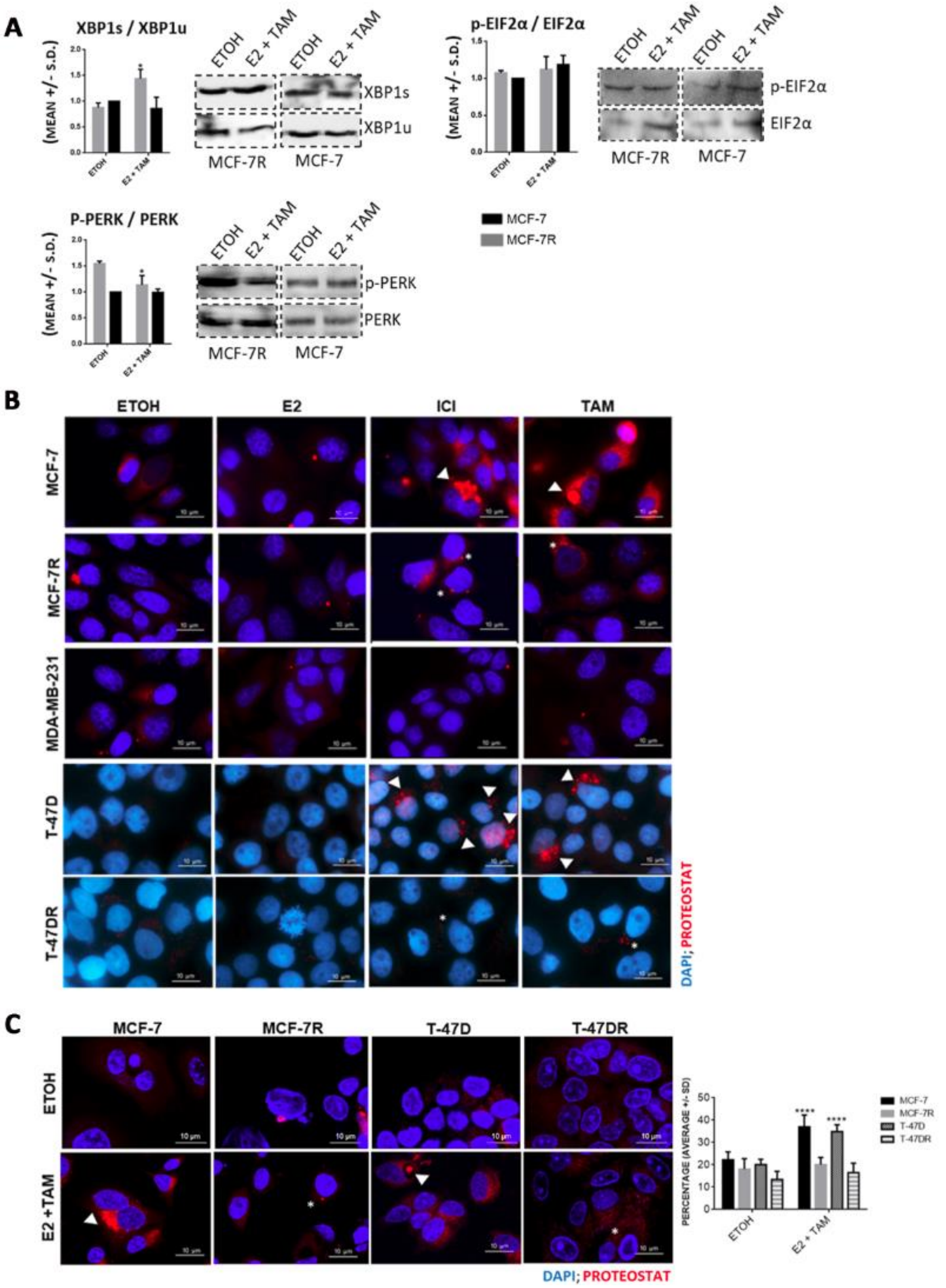

Figure S3. Characterization of antiestrogen-induced protein aggregation clearance and accumulation in breast cancer cell lines. **A.** Western blot showing activation of BIP, XBP1s/XBP1u ratio and PERK/EIF2α expression maintenance after exposure to 10 nM 17β-estradiol (E2) + 500 nM 40HO-tamoxifen (TAM) for 3h. *p<0.05; **p<0.01; ***p<0.001 vs. ETOH in MCF-7 cells. Blots are representative of two experiments. **B.** Comparative analysis of protein aggregation levels in MCF-7, MCF-7R, MDA-MB-231, T-47D and T-47DR cell lines. Staining with Proteostat® was carried out after 24h treatment wiith 10 nM E2, 250 nM ICI, 500 nM TAM. Arrowheads: cytoplasmatic localization; asterix: juxtanuclear region. **C.** Comparative analysis of protein aggregation levels in MCF-7, MCF-7R, T-47D and T-47DR cell lines after 24h treatment wiith 10 nM E2 + 500 nM TAM. The graph summarize results. ****p < 0.0001; vs. same cell line ETOH.

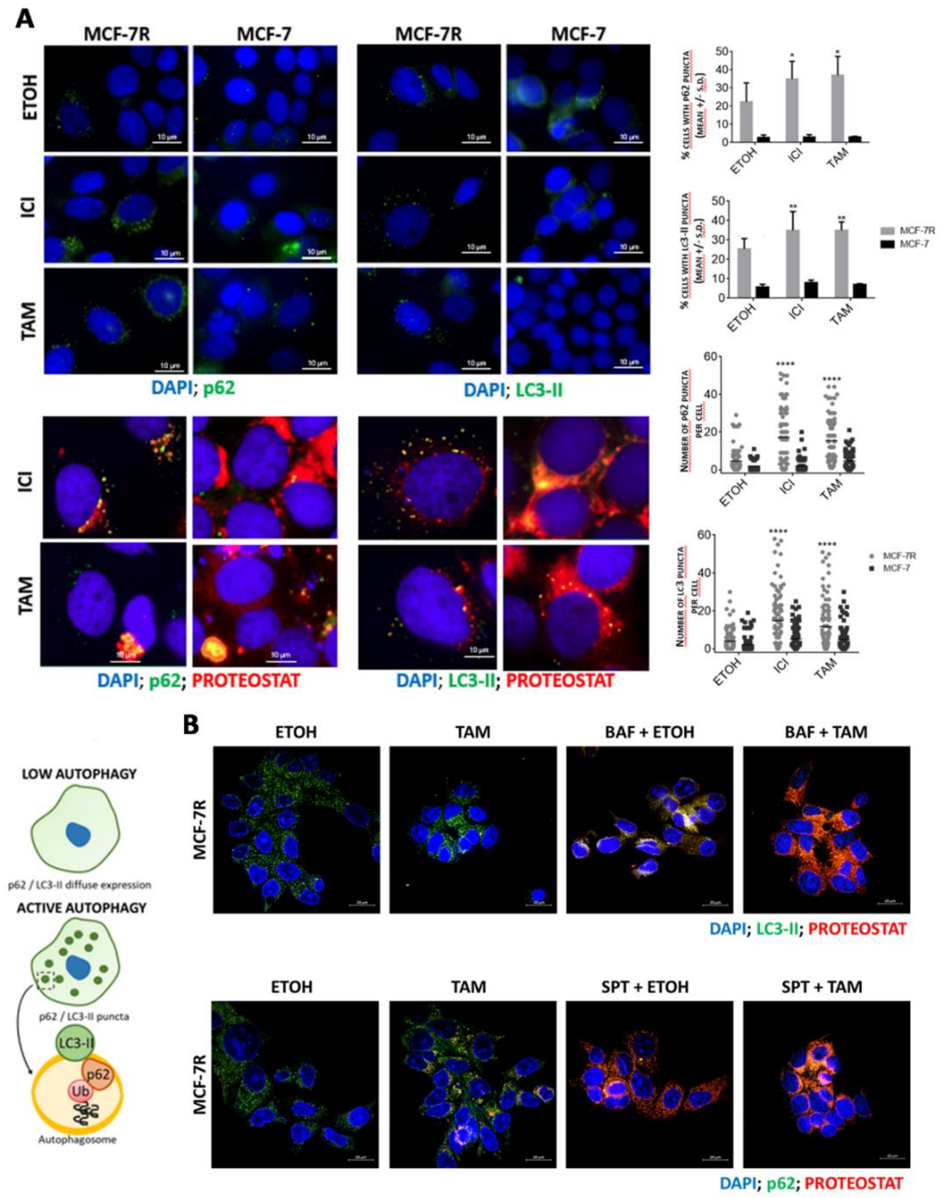

Figure S4. Correlation between autophagy activation and aggresome accumulation following antiestrogen treatment. **A.** MCF-7 and MCF-7R cells were treated with 250 nM ICI, 500 nM TAM or same volume of ETOH (control) for 24h. Subcellular localization of p62 and LC3-II is schematically represented in the cartoon. In a state of low autophagy, p62 (a cargo recognition protein that targets ubiquitinated protein to degradation) and LC3-II (an autophagosome membrane protein) localization is diffuse throughout the cytoplasm. On the other hand, p62/LC3-II puncta expression is a characteristic of active autophagy and can be seen in higher number in MCF-7R cells. The lower panels show p62 and LC3-II co-localization with protein aggregates labelled with ProteoStat®. The pictures depict clear differences in the autophagic patterns between MCF-7 and MCF-7R cells in response to treatment as well as lower co-localization of autophagic markers with aggresomes in MCF-7 cells. N=6000 cells analyzed. In all graphs ****$p < 0.0001$, ***$p < 0.001$, **$p < 0.01$, *$p < 0.05$ vs. same cell line ETOH, non-parametric t test. **B.** MCF-7R were co-incubated with 500 nM tamoxifen (TAM) and 1μM of Bafilomycin (lysosomal inhibitor) or 1μM Spautin-1 (USP10 and USP13 inhibitor) for 8h. Inhibition of autophagy increased protein aggregation and co-localization of LC3-II or p62 with aggresomes.

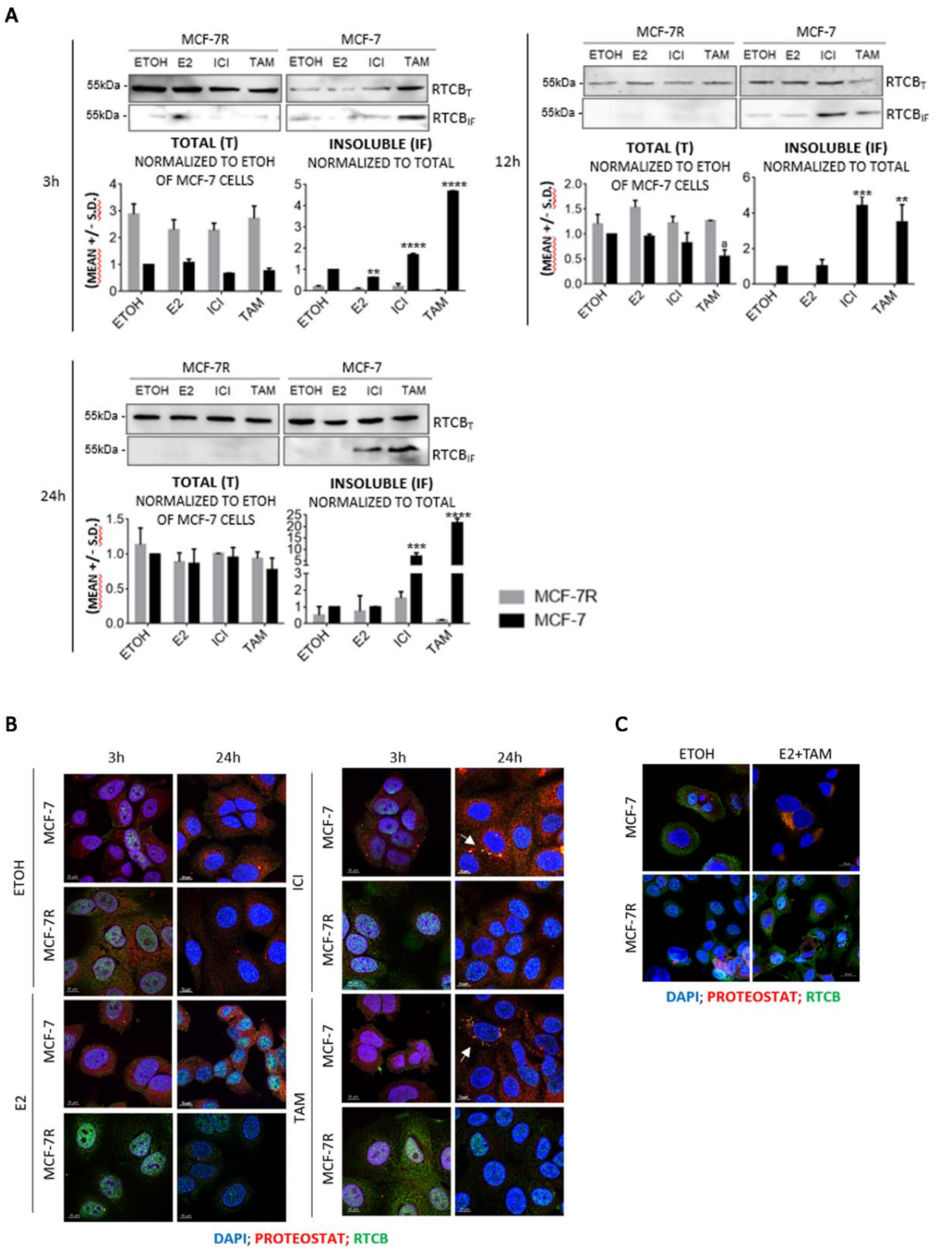

Figure S5. Changes in RTCB expression levels in the total (T) and Insoluble Fractions (IF) of MCF-7 and MCF-7R cells along a period of 24h following ETOH (control), 10 nM E2, 250 nM ICI or 500 nM TAM treatments. **A.** RTCB aggregation was detected in IFs of MCF-7 cells as early as 3h and reached a maximum after 24h of ICI or TAM treatment; Pictures are representative of two independent experiments. In all graphs ****p < 0,0001, ***p < 0,001, **p < 0,01 vs. same cell line ETOH, non-parametric t test. **B.** Double ICC with Proteostat® and RTCB in MCF-7 and MCF-7R cell lines demonstrates that antiestrogen treatment increases RTCB accumulation in aggresomes of sensitive cells. At 3h RTCB is manly localized in the nucleus of MCF-7R cells; decreased nuclear localization of RTCB occurs in parallel with increased RTCB aggregation in MCF-7 cells treated with ICI or TAM; after 24h of endocrine treatment, the number and the size of aggregates containing RTCB increased in MCF-7 cells, as well as the loss of nuclear localization. Pictures are representative of two independent experiments. **C.** Colocalization of RTCB and aggresomes after 24h incubation with 10 nM E2 + 500 nM TAM demonstrates that TAM alone or in combination with E2 produces the same effect, leading to RTCB aggregation only in sensitive cells.

Table S1. Clinicopathological data of the patient cohort.

Case	Age at diagnosis	Histological type	Grade	Stage	Molecular subtype	Adjuvant Treatment	Years until metastasis	Metastasis topography	Disease course	Vital Status
1	36	Mixed	2	III	Luminal B-like, HER2+	CT + RT + Exemestane + Trastuzumab	10	Brain	Disease progression, under endocrine treatment (skin, bone metastases)	Dead of disease
2	51	Ductal	2	II	Luminal-like, HER2-	CT + RT + Anastrazole	3	Liver	Disease progression (bone metastases)	Dead of disease
3	56	Ductal	3	II	Luminal-like, HER2-	CT + RT + Anastrazole	8	Skin	Disease progression (bone metastases)	Dead of disease
4	43	Ductal	2	III	Luminal B-like, HER2+	CT + RT + Tamoxifen + Trastuzumab	5	Lung	Disease progression, under endocrine treatment (pleural and bone metastases)	Dead of disease
5	56	Ductal	2	II	Luminal B-like	CT + RT + Letrozol	19	Lung	Disease progression, under endocrine treatment (bone)	Alive with disease
6	35	Ductal	2	III	Luminal B-like, HER2+	CT + RT + Tamoxifen + Trastuzumab	5	Lung	Disease progression, under endocrine treatment (bone, mediastinal)	Dead of disease
7	41	Mixed	2	II	Luminal-like, HER2-	CT + RT + Tamoxifen	9	Lung	Disease progression, under endocrine treatment (lung metastases)	Dead of disease
8	48	Ductal	3	III	Luminal B-like, HER2-	CT + RT + Tamoxifen	10	Lung	Disease progression, under endocrine treatment (bone metastases)	Alive with disease
9	58	Ductal	3	II	Luminal B-like, HER2+	CT + Anastrazole + Trastuzumab	6	Lung	Disease progression, under endocrine treatment (bone, brain metastases)	Alive with disease
10	74	Ductal	2	I	Luminal-like, HER2-	CT + RT + Tamoxifen	9	Lung	Disease progression, under endocrine treatment (bone, liver metastases)	Dead of disease
11	49	Ductal	2	II	Luminal-like, HER2-	CT + RT + Tamoxifen	12	Lung	Disease progression (pleural metastases, elevated CA15.3)	Alive with disease
12	59	Ductal	3	N/A	Luminal-like, HER2+	CT + RT + Tamoxifen	6	Pleural	Disease progression, under endocrine treatment (bone, skin, lymph-node metastases)	Dead of disease
13	67	Lobular	2	II	Luminal-like, HER2-	CT + RT + Anastrazole	8	Pleural	Disease progression (bone metastases)	Dead of disease
14	68	Lobular	2	II	Luminal-like, HER2-	CT + RT + TamoxifenàExemestane	8	Pleural	Disease progression, under endocrine treatment (bone, liver metastases)	Dead of disease
15	44	Ductal	3	III	Luminal-like, HER2-	CT + RT + Tamoxifen	5	Pleural	Disease progression, under endocrine treatment (bone, brain, liver, lymph-node metastases)	Dead of disease
16	45	Ductal	2	III	Luminal-like, HER2-	CT + RT + Tamoxifen	5	Pleural	Disease progression (bone, lymph-node, liver, skin metastases)	Dead of disease
17	50	Ductal	2	I	Luminal-like, HER2-	CT + RT + TamoxifenàExemestane	7	Pleural	Disease progression, under endocrine treatment (bone metastases)	Dead of disease
18	66	Ductal	3	III	Luminal-like, HER2+	CT + Anastrazole + Trastuzumab	2	Pleural	Disease progression (lung metastases)	Dead of disease
19	54	Ductal	3	I	Luminal-like, HER2-	CT + Anastrazole	5	Pleural	Disease progression, under endocrine treatment (lung, pericardium, bone, lymph-node metastases)	Dead of disease
20	51	Lobular	2	I	Luminal-like, HER2-	CT + Tamoxifen	2	Pleural	Disease progression, under endocrine treatment (bone, liver metastases)	Dead of disease

21	52	Lobular	2	III	Luminal-like, HER2-	CT + RT + Anastrazole	3	Pleural	Disease progression (lung, bone metastases)	Dead of disease
22	69	Ductal	3	III	Luminal-like, HER2-	CT + RT + Anastrazole	4	Pleural	N/A	Died of stage IV gastric cancer

Table S2. Proteins found uniquely aggregated in MCF-7R cells following 24h incubation with 4OH-tamoxifen (TAM) or Fulvestrant (ICI).

**TAM or ICI**			
**Uniprot IDs**	**Entry name**	**Protein name**	**Gene names**
Q9HCC0	MCCB_HUMAN	Methylcrotonoyl-CoA carboxylase beta chain, mitochondrial	MCCC2; MCCB
Q15907	RB11B_HUMAN	Ras-related protein Rab-11B	RAB11B; YPT3
P02545	LMNA_HUMAN	Prelamin-A/C	LMNA; LMN1
P61313	RL15_HUMAN	60S ribosomal protein L15	RPL15; EC45; TCBAP0781
P46783	RS10_HUMAN	40S ribosomal protein S10	RPS10
P23284	PPIB_HUMAN	Peptidyl-prolyl cis-trans isomerase B	PPIB; CYPB
Q07020	RL18_HUMAN	60S ribosomal protein L18	RPL18
Q9NVI7	ATD3A_HUMAN	ATPase family AAA domain-containing protein 3A	ATAD3A
Q03252	LMNB2_HUMAN	Lamin-B2	LMNB2; LMN2
P61247	RS3A_HUMAN	40S ribosomal protein S3a	RPS3A; FTE1; MFTL
P61978	HNRPK_HUMAN	Heterogeneous nuclear ribonucleoprotein K	HNRNPK; HNRPK
P62906	RL10A_HUMAN	60S ribosomal protein L10a	RPL10A; NEDD6
P62424	RL7A_HUMAN	60S ribosomal protein L7a	RPL7A; SURF-3; SURF3
P04899	GNAI2_HUMAN	Guanine nucleotide-binding protein G(i) subunit alpha-2	GNAI2; GNAI2B
Q96QK1	VPS35_HUMAN	Vacuolar protein sorting-associated protein 35	VPS35; MEM3; TCCCTA00141
P48729	KC1A_HUMAN	Casein kinase I isoform alpha	CSNK1A1
P62753	RS6_HUMAN	40S ribosomal protein S6	RPS6; OK/SW-cl.2
Q9BQ70	TCF25_HUMAN	Transcription factor 25	TCF25; KIAA1049; NULP1; FKSG26
P62241	RS8_HUMAN	40S ribosomal protein S8	RPS8; OK/SW-cl.83
P40429	RL13A_HUMAN	60S ribosomal protein L13a	RPL13A
P78527	PRKDC_HUMAN	DNA-dependent protein kinase catalytic subunit	PRKDC; HYRC HYRC1
Q14204	DYHC1_HUMAN	Cytoplasmic dynein 1 heavy chain 1	DYNC1H1; DHC1; DNCH1; DNCL; DNECL; DYHC; KIAA0325
Q9HCE1	MOV10_HUMAN	Helicase MOV-10	MOV10; KIAA1631
Q14151	SAFB2_HUMAN	Scaffold attachment factor B2	SAFB2; KIAA0138
P56192	SYMC_HUMAN	Methionine--tRNA ligase, cytoplasmic	MARS1; MARS
P46940	IQGA1_HUMAN	Ras GTPase-activating-like protein IQGAP1	IQGAP1; KIAA0051
Q8IY81	SPB1_HUMAN	pre-rRNA 2′-O-ribose RNA methyltransferase FTSJ3	FTSJ3; SB92
P33992	MCM5_HUMAN	DNA replication licensing factor MCM5	MCM5; CDC46
Q7Z406	MYH14_HUMAN	Myosin-14	MYH14; KIAA2034; FP17425
P53621	COPA_HUMAN	Coatomer subunit alpha	COPA

Q96EY7	PTCD3_HUMAN	Pentatricopeptide repeat domain-containing protein 3, mitochondrial	PTCD3; MRPS39; TRG15
P49736	MCM2_HUMAN	DNA replication licensing factor MCM2	MCM2; BM28; CCNL1; CDCL1; KIAA0030
P14678	RSMB_HUMAN	Small nuclear ribonucleoprotein-associated proteins B and B′	SNRPB; COD SNRPB1
Q14103	HNRPD_HUMAN	Heterogeneous nuclear ribonucleoprotein D0	HNRNPD; AUF1; HNRPD
Q1KMD3	HNRL2_HUMAN	Heterogeneous nuclear ribonucleoprotein U-like protein 2	HNRNPUL2; HNRPUL2
Q92598	HS105_HUMAN	Heat shock protein 105 kDa	HSPH1; HSP105; HSP110; KIAA0201
Q16891	MIC60_HUMAN	MICOS complex subunit MIC60	IMMT; HMP; MIC60; MINOS2; PIG4; PIG52
Q9UKM9	RALY_HUMAN	RNA-binding protein Raly	RALY; HNRPCL2; P542
Q00839	HNRPU_HUMAN	Heterogeneous nuclear ribonucleoprotein U	HNRNPU; C1orf199; HNRPU; SAFA; U21.1
Q00610	CLH1_HUMAN	Clathrin heavy chain 1	CLTC; CLH17; CLTCL2; KIAA0034
P05141	ADT2_HUMAN	ADP/ATP translocase 2	SLC25A5; ANT2
Q15424	SAFB1_HUMAN	Scaffold attachment factor B1	SAFB; HAP; HET; SAFB1
Q99623	PHB2_HUMAN	Prohibitin-2	PHB2; BAP; REA
Q15020	SART3_HUMAN	Squamous cell carcinoma antigen recognized by T-cells 3	SART3; KIAA0156; TIP110
P43243	MATR3_HUMAN	Matrin-3	MATR3; KIAA0723
Q96JB2	COG3_HUMAN	Conserved oligomeric Golgi complex subunit 3	COG3; SEC34
P51398	RT29_HUMAN	28S ribosomal protein S29, mitochondrial	DAP3; MRPS29
Q9H993	ARMT1_HUMAN	Damage-control phosphatase ARMT1	ARMT1; C6orf211
Q9NVP1	DDX18_HUMAN	ATP-dependent RNA helicase DDX18	DDX18; cPERP-D
O75396	SC22B_HUMAN	Vesicle-trafficking protein SEC22b	SEC22B; SEC22L1
O75340	PDCD6_HUMAN	Programmed cell death protein 6	PDCD6; ALG2
P42766	RL35_HUMAN	60S ribosomal protein L35	RPL35
P14866	HNRPL_HUMAN	Heterogeneous nuclear ribonucleoprotein L	HNRNPL; HNRPL; P/OKcl.14
Q92841	DDX17_HUMAN	Probable ATP-dependent RNA helicase DDX17	DDX17
P53007	TXTP_HUMAN	Tricarboxylate transport protein, mitochondrial	SLC25A1; SLC20A3
P35268	RL22_HUMAN	60S ribosomal protein L22	RPL22
Q92616	GCN1_HUMAN	eIF-2-alpha kinase activator GCN1	GCN1; GCN1L1; KIAA0219
Q8WUM4	PDC6I_HUMAN	Programmed cell death 6-interacting protein	PDCD6IP; AIP1; ALIX; KIAA1375
O94832	MYO1D_HUMAN	Unconventional myosin-Id	MYO1D KIAA0727
P84098	RL19_HUMAN	60S ribosomal protein L19	RPL19
P36542	ATPG_HUMAN	ATP synthase subunit gamma, mitochondrial	ATP5F1C; ATP5C; ATP5C1; ATP5CL1
